# Multifunctional E‐Tattoos Based on Electrospun PVBVA Fibers Coated with Ti_3_C_2_T*_x_* MXene for Energy Harvesting, Energy Storage, and Biometric Sensing

**DOI:** 10.1002/advs.202518697

**Published:** 2025-12-07

**Authors:** Ajay Pratap, Fereshteh Rajabi Kouchi, Tony Valayil‐Varghese, Hailey Burgoyne, Attila Rektor, Michael Curtis, Miranda Lea Nelson, Francis N. Mokogwu, Corey M. Efaw, Josh Eixenberger, Allyssa Bateman, Benjamin C. Johnson, Brian Jaques, Zhangxian Deng, Kurtis Cantley, Christopher E. Shuck, David Estrada

**Affiliations:** ^1^ Micron School of Material Science and Engineering Boise State University Boise ID 83725 USA; ^2^ Micron Center for Materials Research Boise State University Boise ID 83725 USA; ^3^ Biomedical Engineering Doctoral Program Boise State University Boise ID 83725 USA; ^4^ Department of Electrical and Computer Engineering Boise State University Boise ID 83725 USA; ^5^ Department of Physics Boise State University Boise ID 83725 USA; ^6^ Center for Advanced Energy Studies Boise State University Boise ID 83725 USA; ^7^ Mechanical and Biomedical Engineering Boise State University Boise ID 83725 USA; ^8^ Department of Chemistry and Chemical Biology Rutgers University Piscataway NJ 08854 USA; ^9^ Idaho National Laboratory Idaho Falls ID 83415 USA

**Keywords:** electrospinning, energy harvesting, energy‐storage, E‐tattoo, MXene

## Abstract

Multifunctional electronic tattoos (e‐tattoos) integrating energy harvesting, charge storage, and biosignal monitoring are critical for advancing wearable electronics. Most current technologies specialize in one or two functions, lacking a unified, skin‐compatible solution. A novel e‐tattoo is reported using electrospun poly(vinyl butyral‐co‐vinyl alcohol‐co‐vinyl acetate) (PVBVA) fibers coated with titanium carbide (Ti_3_C_2_T*_x_*) MXene. A single electrode triboelectric nanogenerator (TENG) was fabricated via a layer‐by‐layer method using a PVBVA/Ti_3_C_2_T*_x_*/PVBVA (PMxP) sandwich structure, and achieved an open‐circuit voltage of 250 V, short‐circuit current of 2.9 µA, and power density of 250 mW m^−^^2^ (25 µW cm^−^^2^) under a 2 MΩ load, enabling triboelectric energy harvesting from human motion. A parallel‐plate capacitor using PVBVA/Ti_3_C_2_T_*x*_ electrodes and a PVBVA dielectric exhibited 14 pF capacitance at 10 kHz and 5 V, suitable for low‐power touch‐sensitive applications. Additionally, PMx‐based e‐tattoos captured real‐time electrocadiogram (ECG) and electromyography (EMG) signals with high skin conformability and minimal signal degradation. The device maintains mechanical flexibility, biocompatibility, and adhesion over extended wear. This scalable, non‐invasive platform demonstrates robust multifunctionality and durability, offering a promising route toward integrated, self‐powered wearable systems for health monitoring, human‐machine interfaces, and energy autonomy. The PMxP architecture represents a significant step toward all‐in‐one e‐tattoos that meet the demands of next‐generation electronics.

## Graphical abstract

This study explores a novel E‐tattoo made from PVBVA fibers coated with Ti_3_C_2_T_*x*_ MXene. The device is designed to harvest energy directly from the human body, providing power for itself. The research demonstrates the E‐tattoo's capability for charge storage and its potential for health monitoring through integrated ECG and EMG sensing, all within a single system that adheres directly to the skin.

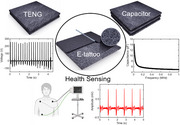

## 1 Introduction

The advent of electronic tattoos (e‐tattoos)^[^ ^1^ ^]^ has contributed to a transformative period for applications in health monitoring,^[^ ^2^ ^]^ drug delivery,^[^ ^3^ ^]^ energy harvesting,^[^ ^4^ ^]^ and human‐machine interfaces (HMIs).^[^ ^4^ ^]^ A pioneering contribution in this field was made by Rogers et al.,^[^ ^5^, ^6^, ^7^ ^]^ who developed an ultra‐thin, skin‐conformal electronic system capable of adhering directly to the epidermis without the need for conventional adhesives or gels. Their system, constructed on a polyester substrate and integrated with embedded sensors, demonstrated high sensitivity and mechanical compliance to skin deformation. The use of polyvinyl alcohol (PVA) as a temporary transfer layer introduced complexity to the fabrication process and compromised long‐term device stability and seamless skin integration. The reliance on sophisticated microfabrication techniques, such as photolithography, to define conductive pathways increased fabrication cost and complexity. The non‐porous nature of the polyester substrate inhibited skin transpiration, limiting comfort and usability for prolonged wear.^[^ ^5^ ^]^ These limitations highlight the need for next‐generation electronic skin (e‐skin) platforms that can waive transfer layers, offer intrinsic breathability, and maintain strong skin adhesion, biocompatibility, and mechanical durability for extended use.

To overcome integration and fabrication limitations of traditional processes, techniques such as drop casting,^[^ ^8^ ^]^ spin‐coating^[^ ^9^ ^]^ and electrospinning^[^ ^10^ ^]^ are being utilized. Electrospinning has emerged as a promising strategy for developing breathable, porous, and skin‐conformal substrates suitable for wearable electronics.^[^ ^11^ ^]^ Bao^[^ ^12^, ^13^ ^]^ pioneered the use of electrospinning in the context of e‐skin systems, demonstrating its ability to yield ultra‐thin, flexible platforms for real‐life physiological sensing applications. Electrospinning facilitates the direct formation of fibrous mats that are not only mechanically robust and lightweight but also adhere naturally to the contours of the skin without requiring additional adhesives or transfer steps. Over the years, a wide array of polymers and functional materials, including BaTiO_3_, PVDF,^[^ ^14^ ^]^ PEO,^[^ ^15^ ^]^ and PVA^[^ ^16^ ^]^ have been explored to impart specific electrical, mechanical, or piezoelectric properties to these fibrous networks. In the domain of biomaterials, Narender et al. introduced a silk‐based electrospun fiber platform,^[^ ^11^, ^15^ ^]^ offering good breathability, skin conformity, and multifunctional sensing performance. Despite its biocompatibility, silk presents inherent limitations: it cannot form fibers independently and must be blended with a carrier polymer such as polyethylene oxide (PEO), and its water solubility undermines long‐term reliability in humid environments. These constraints underscore the critical need for alternative materials that seamlessly combine electro‐spinnability, mechanical integrity, environmental stability, and biocompatibility into a single platform for next‐generation skin‐interfaced electronics.

One of the most pressing challenges in the advancement of wearable electronics is the continuous need for power, which traditionally relies on bulky batteries^[^ ^17^, ^18^ ^]^ or rigid power modules that hinder device miniaturization, long‐term operation, and overall user comfort. While conventional energy storage solutions like lithium‐ion batteries offer high energy density,^[^ ^19^ ^]^ they are often rigid, non‐breathable, and pose safety concerns due to risks of leakage or thermal runaway in skin‐mounted configurations. Alternative energy‐harvesting mechanisms such as piezoelectricity,^[^ ^20^ ^]^ thermoelectricity,^[^ ^21^, ^22^ ^]^ triboelectricity^[^ ^23^ ^]^ and electromagnetic induction^[^ ^24^ ^]^ have been explored for wearable applications. Among these, triboelectric nanogenerators (TENGs) have gained particular attention for their ability to convert low‐frequency biomechanical motion into electrical energy in a lightweight and compact format.^[^ ^25^, ^26^ ^]^ TENGs are inherently well‐suited for harvesting energy from daily human activities, enabling the development of self‐powered wearable systems. Many current TENG architectures rely on separate triboelectric layers such as PDMS (Polydimethylsiloxane), nitrile, or metallic film materials that increase device thickness and reduce skin conformity.^[^ ^27^ ^]^ This lack of seamless integration between the energy‐harvesting element and the wearable substrate continues to limit the performance and practicality of TENGs in long‐term, on‐skin applications.

Intermittent power output from energy harvesters presents challenges for devices requiring consistent power. Conventional capacitors, used for temporary energy storage and power conditioning, are rigid or bulky, complicating system design and reducing platform flexibility. This lack of integrated energy storage restricts autonomous function in wearable electronics, especially where consistent power is essential, such as bio‐signal monitoring. Researchers have shown thin‐film capacitors or supercapacitors with materials like tungsten oxide (WO_3_), which have high theoretical capacity.^[^ ^28^ ^]^ Recently, Rajabi‐Kouchi et al. demonstrated a fully printed supercapacitor using MXene inks, highlighting the potential of these materials in additive manufacturing of energy storage systems.^[^ ^29^ ^]^ Seamless energy storage is crucial for electronic skin systems for health measurements like electrocardiogram (ECG) and electromyography (EMG) signal capture. Current sensor configurations are typically hard‐wired to an external power source and use conductive gels, rigid electrodes, or adhesive interfaces, which cause skin irritation and limit long‐term stability and comfort.^[^ ^30^ ^]^

There is a growing need for integrated systems with harvesting, charge storage, and biosensing, while maintaining skin‐conformability, breathability, and biocompatibility. Towards this end, research demonstrates electrospun fiber utilization in textile‐based integrated devices for wearable health monitoring, energy harvesting, and charge storage. Zhi et al. have recently advanced the field by fabricating a self‐powered, biocompatible, and antibacterial textile‐based TENG capable of tactile sensing.^[^ ^31^ ^]^ Shalik et al. have also studied melanin‐doped silk nanofibers (SNFs) and graphene, demonstrating their utility for skin hydration monitoring, UV detection, respiration sensing, and low‐noise electrophysiological signal measurement, like ECG.^[^ ^32^ ^]^ Their work demonstrates that integrating triboelectric functions within textile substrates can lead to promising wearable systems, but several challenges remain unaddressed. These include difficulties in ensuring long‐term mechanical durability under real‐life conditions like repeated deformation, limitations in seamless integration with the human body, and scalability issues in manufacturing. Their system still relied on external textile layers, which restrict intimate skin integration. A platform that inherently integrates these functionalities would simplify device architecture and enhance practical utility for next‐generation wearable applications.

Here, we present the development of a highly flexible and mechanically stable e‐tattoo, fabricated using electrospun poly(*vinyl butyral‐co‐vinyl alcohol‐co‐vinyl acetate*) (PVBVA) fibers coated with Ti_3_C_2_T_*x*_ MXene. MXenes are an emerging class of 2D nanomaterials with good electrical conductivity and mechanical robustness.^[^ ^33^, ^34^, ^35^ ^]^ MXene's stable, sheet‐like structure makes them highly suitable for integration into electrospun platforms for various electronic and sensing applications.^[^ ^36^, ^37^ ^]^ The resulting device exhibits good skin conformity and can be easily integrated onto the human body for a wide range of applications. As a demonstration of its multifunctionality, we first fabricated a TENG for mechanical energy harvesting. The device achieved a peak output of 250 V and 2.9 µA, with a corresponding power density of 250 mW m^−^^2^ at an external load of 2 MΩ, confirming its potential as a self‐sufficient power source. We also developed a self‐powered touch sensor based on the same platform, suitable for various interactive and sensing applications. A 3 × 3 matrix sensor array was also fabricated, enabling the demonstration of a human–machine interface (HMI) system. To explore the e‐tattoo's energy storage capability, we fabricated a parallel plate capacitor, which exhibited a capacitance of 14 pF at a frequency of 10 kHz under an applied bias of 5 V, indicating its feasibility for transient energy storage. Owing to its good skin adhesion and biocompatibility, the e‐tattoo was evaluated for health monitoring applications. Real‐time recordings of both electrocardiogram (ECG) and electromyography (EMG) signals were successfully obtained, highlighting its effectiveness as a wearable biosensor.

## 2 Results and Discussion

**Figure 1:**
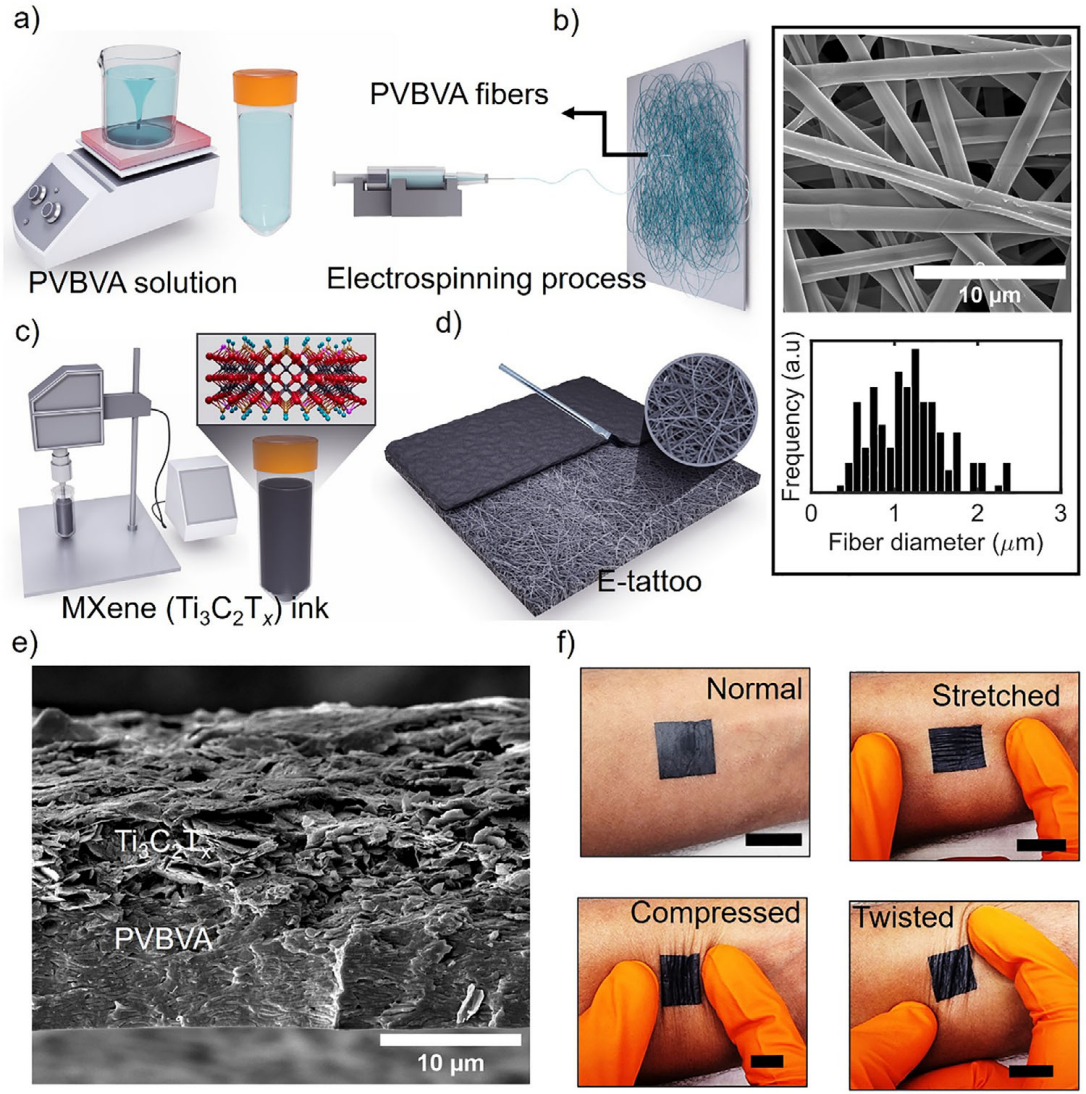
Schematic and characterization of the preparation of PVBVA/MXene‐based e‐tattoo. a) Preparation of PVBVA solution for electrospinning b) Electrospinning process for PVBVA fibers. The SEM image of the fibers, highlighting their morphology and diameter distribution c) Preparation of Ti_3_C_2_T*_x_* MXene ink d) Fabrication process of the e‐tattoo, demonstrating its structural configuration e) SEM cross‐sectional image of the fabricated e‐tattoo, revealing the layered microstructure and fiber‐matrix interactions f) Mechanical adaptability of the e‐tattoo, showing stable attachment under various deformations, including normal, stretched, compressed, and twisted states (Scale bar 2 cm).

**Figure** **1**a–d illustrates the detailed fabrication process of the e‐tattoo based on electro‐spun PVBVA fibers and painting of Ti_3_C_2_T*_x_* to make an electrically conductive layer. The initial step involves preparing a solution for electrospinning. This was achieved by dissolving 5 wt.% of PVBVA nanoparticles in ethanol. We mixed 2 g of PVBVA nanoparticles in 40 mL of ethanol, heated at 60 °C, and stirred for 4 h to make a homogeneous solution. The prepared PVBVA solution was then loaded into a syringe and attached to the electrospinning machine (Figure 1a). Through the electrospinning process, fibers are formed from the PVBVA solution on the aluminum foil, which is attached to a vertical substrate with scotch tape. To ascertain the orientation and diameter of the obtained fibers, scanning electron microscopy (SEM) was employed, followed by the utilization of ImageJ software for diameter quantification. The SEM image shows the morphology and orientation of the PVBVA fiber in Figure 1b. Subsequent measurements conducted using ImageJ revealed that the diameter of the fibers ranged from 0.8–1.2 µm. For the conductive layer, we prepared a multilayer Ti_3_C_2_T*_x_* ink (Figure 1c). The detailed parameters and conditions of the electrospinning process and Ti_3_C_2_T*_x_* ink preparations with characterizations can be seen in the methods section. To fabricate an e‐tattoo, a simple paintbrush was used to coat Ti_3_C_2_T*_x_* ink on the fibers (Figure 1d). The sample was then heated inside an argon glovebox at 40–50 °C for at least 24 h to evaporate the solvent. Although the ink formulation includes ethylene glycol (EG), which is known to be toxic in its liquid form, several factors ensure the biocompatibility of the final device. The EG is used in combination with ethanol to aid the dispersion and film‐forming quality of the Ti_3_C_2_T_x_ MXene ink. Following deposition, the extended thermal treatment allows sufficient time for solvent evaporation. Despite EG's relatively low vapor pressure compared to ethanol, its hygroscopic nature and partial miscibility with ethanol facilitate co‐evaporation during the drying process.^[^ ^38^, ^39^, ^40^ ^]^ In order to establish the e‐tattoo's biocompatibility, we conducted a cytotoxicity assay, the complete findings and discussion of which are provided later in this document. After solvent removal, the MXene layer becomes physically confined within the porous PVBVA fiber network, which reduces the risk of direct skin exposure to any potential residuals. During subsequent handling and ambient storage, additional solvent evaporation is likely to occur, further minimizing any remaining traces, and no signs of irritation or adverse reactions were observed during real‐time on‐skin applications in any measurements, indicating that the processed e‐tattoos are safe for epidermal use. These observations collectively support the effective removal of toxic solvents and the overall biocompatibility of the e‐tattoo platform. The fabricated e‐tattoo was conductive (Figure S1, Supporting Information), permeable, breathable, and conformably attached to the human skin without any adhesive. The e‐tattoo has a dimension of 1 cm × 1 cm and a thickness of ≈20 µm, as shown in the SEM image (Figure 1e). To facilitate conformal attachment, a small amount of DI water was applied to the skin prior affixing the e‐tattoo. Although PVBVA is intrinsically hydrophobic,^[^ ^41^ ^]^ the temporary presence of moisture enhances surface contact by improving adhesion through Van der Waals interactions and allowing the porous fiber mat to conform closely to the skin's microtextured surface. Once attached, the water rapidly evaporates, leaving the tattoo firmly in place. The e‐tattoo itself is lightweight, weighing ≈0.6 mg (Figure S2, Supporting Information), and this light structure, combined with strong skin adhesion, provides good mechanical stability and resistance to deformation, even during dynamic motion. These characteristics are illustrated in Figure 1f, showing the e‐tattoo's wearability and functional performance under various physical conditions, including stretching, compressing, and twisting. To evaluate the breathability of the material, we conducted a water vapor transmission test. Four glass bottles were each filled with a fixed amount of water; three were sealed with different membranes, PDMS, electrospun PVBVA fibers, and the PVBVA/MXene e‐tattoo, while one bottle was left open as a reference. The samples were placed in a controlled environment (30% relative humidity and 21 °C), and the water loss (in mg) was recorded at various time intervals to calculate the water vapor transmission rate (WVTR) (Figure S3, Supporting Information). For a more detailed assessment, small bottles containing 800 mg of water were sealed with the following membranes: no membrane (control), a single PVBVA fiber layer (16 µm thick), the e‐tattoo (20 µm thick), and PDMS (550 µm thick). To reduce the influence of thickness, the WVTR was normalized by the film thickness (l× WVTR). The results showed that the e‐tattoo exhibited a WVTR of 1038.68 g m^−2^ d^−1^, slightly lower than that of the single fiber layer (1054.91 g m^−2^ d^−1^) due to the presence of the MXene layer, but higher than that of the PDMS film (Figure S4, Supporting Information).

The Van der Waals interaction is fundamental to the seamless attachment of PVBVA fibers to skin in the fabricated e‐tattoo.^[^ ^16^, ^17^ ^]^ These weak intermolecular forces enable the e‐tattoo to adhere closely to the skin's surface without requiring robust chemical bonding, ensuring both stability and ease of removal. This mechanism mirrors the Van der Waals bonding described in the context of GaAs epitaxial liftoff films,^[^ ^36^, ^42^ ^]^ where thin films are temporarily affixed to substrates through weak dispersive forces, allowing for detachable yet reliable adhesion. Similarly, the e‐tattoo leverages these interactions to maintain a secure yet reversible connection to the skin. The e‐tattoo stands out for its straightforward removal process, which is effectively accomplished using ethanol rather than water (Movie S1, Supporting Information). This behavior is not governed by surface tension alone, but primarily by the chemical affinity between the solvent and the tattoo's components. While water has high surface tension and poor compatibility with the hydrophobic PVBVA matrix, ethanol not only exhibits a lower surface tension but also serves as an organic solvent capable of interacting with and partially dissolving both the PVBVA fibers and Ti_3_C_2_T_x_ MXene. As a result, ethanol readily penetrates the tattoo's matrix, weakening its structural integrity and enabling easy detachment from the skin. In contrast, water is unable to disrupt the adhesive or cohesive forces within the hydrophobic polymer network, leaving the tattoo intact. This selective solubility ensures gentle, complete removal without irritation, making the e‐tattoo ideal for temporary applications requiring reliable adhesion and clean detachment.

**Figure 2:**
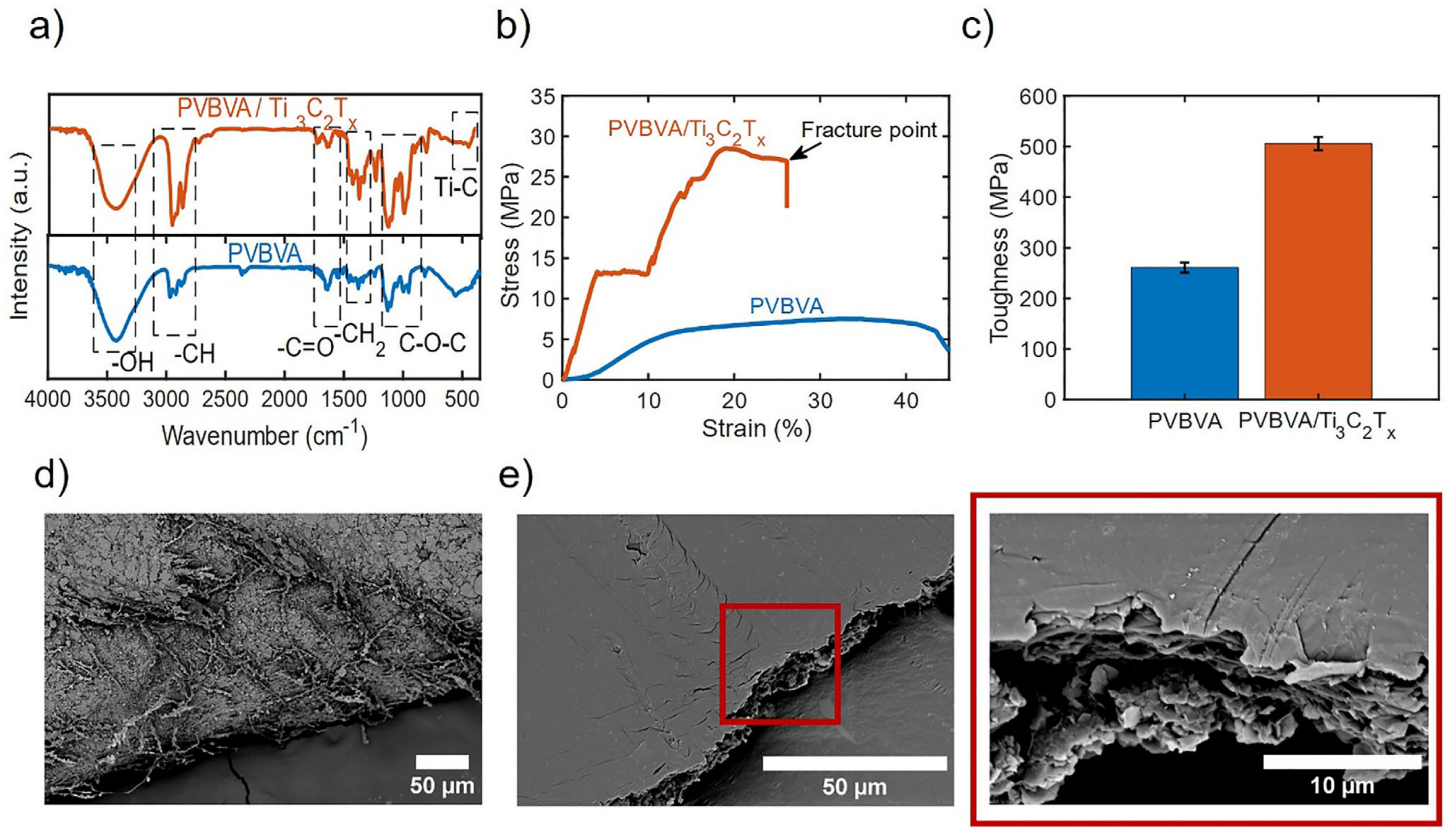
Mechanical and chemical properties of PVBVA fibers and PVBVA/ Ti_3_C_2_T*_x_* composite. a) FTIR spectra of PVBVA and PVBVA/Ti_3_C_2_T*_x_*, highlighting characteristic functional groups b) Stress versus strain analysis of PVBVA and PVBVA/Ti_3_C_2_T*_x_*, showing the fracture point c) Toughness measurements of PVBVA and PVBVA/Ti_3_C_2_T*_x_* (error bar showing standard deviation (SD) of 4 samples) d) SEM image of the PVBVA/Ti_3_C_2_T*_x_* bottom fiber layer after fracture e) SEM image of the top MXene layer with a zoomed‐in view, showing the microstructure.

PVBVA is recognized for its biocompatibility, comprising a polymer matrix formed from vinyl butyral, vinyl acetate, and alcohol units.^[^ ^43^ ^]^ Its distinctive bonding structure, which incorporates both covalent and hydrogen bonds, enhances its mechanical robustness and expands its applicability in various areas. The chemical properties of PVBVA fibers and the PMx (e‐tattoo) composite were analyzed using Fourier transform infrared spectroscopy (FTIR) (**Figure** **2**a). The FTIR spectra revealed key absorption bands that characterize the structural composition of PVBVA fibers. Notably, a peak ≈3430 cm^−1^ corresponds to hydroxyl groups (‐OH), which participate in extensive intermolecular and intramolecular hydrogen bonding. Absorption peaks at 2950 and 2870 cm^−1^ were attributed to asymmetric ─CH stretching vibrations, while peaks at 1640, 1380, 1130, and 1000 cm^−1^ correspond to C═O stretching, CH_2_ bending, and C─O─C stretching vibrations, respectively. For the PMx composite, these absorption peaks were observed but showed reduced intensity, indicating the retention of the polymer's molecular structures. A new peak at 457 cm^−1^ was also observed, that attributed to the Ti─C bond,^[^ ^44^ ^]^ confirming the incorporation of Ti_3_C_2_T_x_ into the composite. The network of covalent and group that can participate in hydrogen bonding within the PVBVA fibers contributes significantly to the material's mechanical stability and toughness, highlighting its potential for diverse applications, particularly in flexible electronics and biocompatible devices.

A coating of Ti_3_C_2_T*_x_* on PVBVA fibers resulted in an improved mechanical performance for the composite material. To validate this enhancement, two samples were prepared for comparison: PVBVA fibers and PMx composite, The analysis is presented in Figure S5 (Supporting Information). The stress–strain analysis (Figure 2b) highlights the significant improvement in the mechanical behavior achieved through the integration of Ti_3_C_2_T*_x_*. The inclusion of this 2D Ti_3_C_2_T*_x_* substantially enhances both the stiffness and tensile strength of the composite compared to PVBVA fibers. The stress–strain curve exhibits an initial linear elastic region, followed by a transition to material failure at a critical stress point. This enhancement is primarily attributed to the porous structure of the PVBVA nanofiber, as shown in Figure 2c, which facilitates effective integration of Ti_3_C_2_T*_x_* ink, thereby improving the material's toughness. A distinct stress–strain behavior was observed for the PMx composite compared to conventional fiber‐based electronic skin. The composite exhibits an elastic response up to a 5% strain, followed by a plateau in stress upon further straining, and then a resurgence of elastic characteristics beyond 10% strain. The toughness (the ability of a material to withstand deformation before fracture) of the composite increased nearly twofold after Ti_3_C_2_T*_x_* integration. The PVBVA fibers exhibit a toughness of ≈260±10 MPa, while the PMx composite reached 513±13 MPa (Figure 2c). The improved elasticity and toughness of the composite can be directly attributed to the presence of Ti_3_C_2_T_*x*_ layers. To quantify this enhancement, we calculated the linear modulus of the PVBVA fiber layers both before and after incorporating MXene using the dots‐to‐plot analysis method. Our findings reveal a significant increase in stiffness, where the bare PVBVA fibers exhibited a linear modulus of 0.667, which increased to 4.373 after MXene incorporation (Figures S6 and S7, Supporting Information). Beyond the linear modulus, we also evaluated other key mechanical properties, including yield stress and strain, ultimate stress, strain at failure, energy absorption capacity, and adhesion of the MXenes to the PVBVA. The comparative results are summarized in Figures S6–S8 (Supporting Information), highlighting the substantial mechanical reinforcement achieved through MXene integration. This enhancement makes the composite more robust and suitable for applications requiring durable and flexible materials. The SEM image shows the upper side (fibers) and the lower side, the bottom (Ti_3_C_2_T*_x_* layer) (Figure 2d,e). These SEM images show how the fibers and Ti_3_C_2_T*_x_* layer contribute effectively to the strength of the e‐tattoo. In Figure 2e the cross‐sectional SEM image reveals noticeable cracks within the Ti_3_C_2_T_x_ MXene layer, which can be attributed to its metal‐like behavior under localized high strain. This behavior contributes to the enhanced toughness and mechanical resilience of the e‐tattoo composite. The presence of metallic Ti–C bonding and various surface terminations in the MXene structure disrupts uniform crack propagation, causing deviations in crack paths. This results in a more irregular and rough fracture surface, as observed in the image. The terraced morphology suggests interlayer sliding and delamination a characteristic failure mechanism commonly seen in layered 2D materials such as multilayer graphene.^[^ ^45^, ^46^ ^]^ These features collectively indicate that MXene not only improves conductivity but also plays a significant role in reinforcing the mechanical structure of the composite e‐tattoo. Although a complete study of the mechanical behavior is in progress. This study highlights the potential of PVBVA and its Ti_3_C_2_T*_x_* composite in fortifying the structural integrity of e‐tattoos, demonstrating their suitability for human‐machine interfaces, especially under varied mechanical deformations. Such mechanical resilience is not only crucial for long‐term wearability but also directly benefits energy harvesting performance in dynamic, skin‐mounted applications. Pratap et al. have previously demonstrated the utility of PVBVA and MXene composites in energy harvesting and sensing contexts, further supporting their integration in multifunctional wearable systems.^[^ ^41^ ^]^

**Figure 3:**
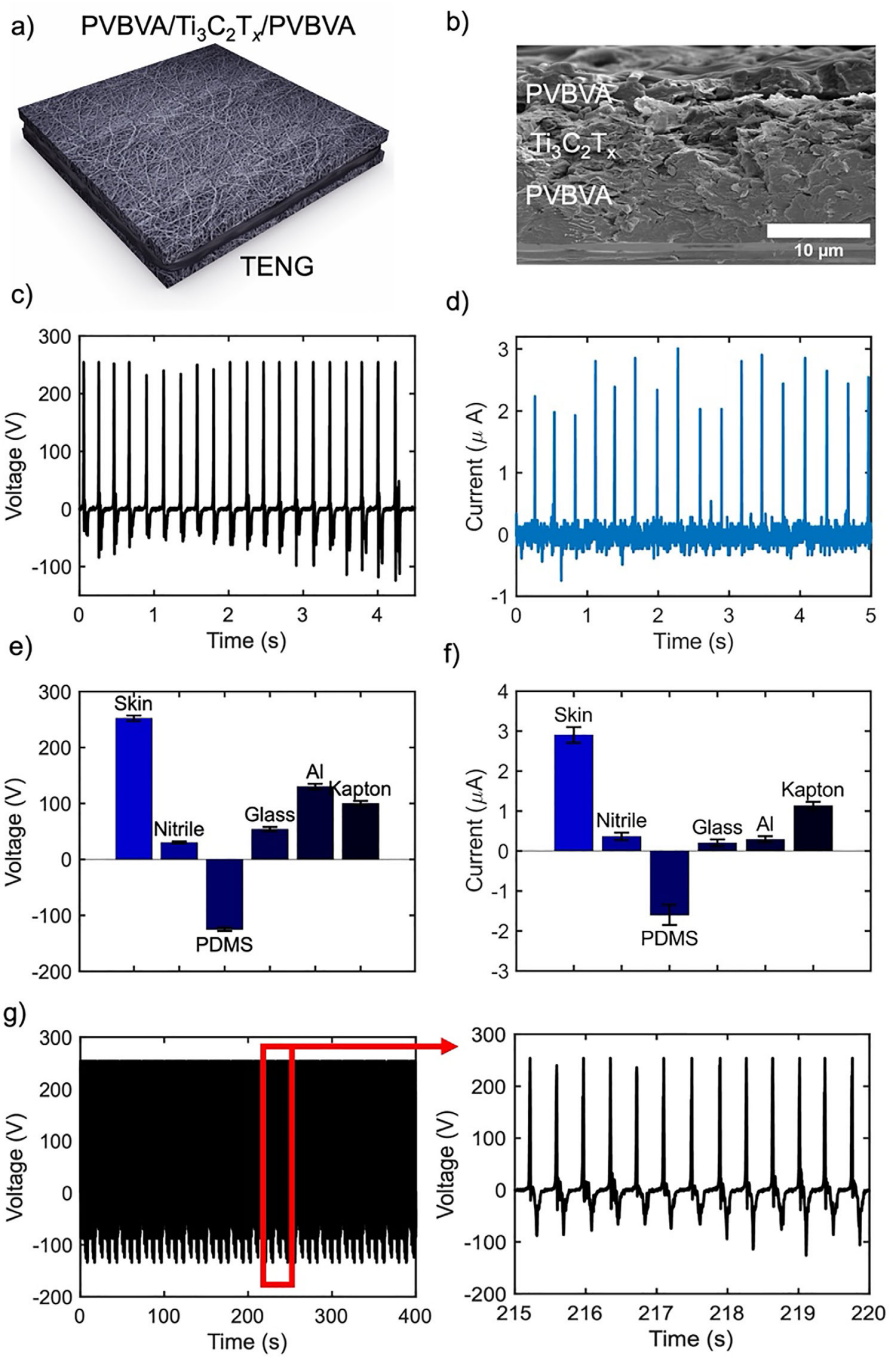
Triboelectric performance of the fabricated single‐electrode‐based triboelectric nanogenerator (TENG). a) Schematic representation of the TENG device, highlighting its sandwich design b) Scanning electron microscope (SEM) image of the TENG's cross section c) Open‐circuit voltage output of the TENG when activated by human skin contact, indicating the voltage generated during operation d) Short‐circuit current output of the TENG under similar activation, illustrating the current produced e,f) Comparison of the TENG's performance with different triboelectric materials, assessing variations in output based on material selection (error bar represents SD of 3 samples) e) Voltage, f) Current g) Mechanical durability test results of the TENG over 3000 cycles, demonstrating the device's stability and longevity.

The development of a innovative e‐tattoo TENGs marks a significant advancement in the field of mechanical energy harvesting. This device, with its unique PMxP structure, is designed to efficiently capture energy from various mechanical sources. The fabrication of the e‐tattoo TENG is illustrated in **Figure** **3**a. The details can be seen via an SEM image (Figure 3b), highlighting that the MXene has been sandwiched between two layers of PVBVA for direct contact with the skin. This design plays a crucial role in the energy harvesting mechanism, as depicted in Figure S9 (Supporting Information), which outlines the operation of the single‐electrode TENG. Initially, when the skin and the e‐tattoo are in equilibrium, there is no induced charge, resulting in an output voltage (V*_oc_*) of zero. As the skin moves away from the PVBVA layer, a charge is induced due to the separation, leading to the generation of an opposing charge on the Ti_3_C_2_T*_x_* layer. This induced charge is collected on a load resistor for potential applications. The process of charge induction results in an output voltage and current that reverse as the finger moves back toward the PVBVA layer, returning the system to its equilibrium state. This cyclical process of charge induction and reversal underlies the TENG's ability to continuously generate power. To explore the triboelectric behavior and interaction between two different materials, skin and PVBVA, we analyzed their inherent surface charge properties. Skin is a tribopositive material that tends to lose electrons, and PVBVA is a tribonegative material that tends to gain electrons. PVBVA exhibits a surface charge potential of ‐5.04 eV, as measured using Scanning Kelvin Probe Force Microscopy (SKPFM) (Figure S10, Supporting Information). This surface potential difference is critical for understanding the charge transfer dynamics in contact electrification. The electron transfer mechanism between skin and PVBVA can be effectively described using the Potential Well Model for contact electrification^[^ ^25^ ^]^ (Figure S11, Supporting Information). According to this model, a tribopositive material has a higher surface potential, meaning it contains more electrons in energy states above the Fermi level, and tribonegative polymer has a lower Fermi level and therefore acts as an electron acceptor. The surface potential of skin (higher) and PVBVA (lower) initially differ. Upon contact, electrons flow from the electron‐rich well to PVBVA to equalize the energy levels. This electron transfer leaves behind positive charges (holes) on the skin and accumulates negative charges on PVBVA. The output voltage (V*_oc_*) and short circuit current (I_*sc*_) of the TENG is calculated using the following (Equations 1 and 2, respectively^[^ ^47^ ^]^)

(1) $$V_{oc}=\frac{-\sigma_{0}A}{2C_{0}}\;$$

(2) $$I_{sc}=\frac{A\;d(\sigma_{0})}{dt}\;$$

where *V_oc_* represents the output voltage, σ_0_ is the induced charge density, *A* is the effective contact area, *C*_0_ is the capacitance of the TENG, I_*sc*_ is the short circuit current, and *dt* is the rate of change of surface charge density. The experimental voltage achieved by the e‐tattoo TENG was ≈250 V (Figure 3c), and the short circuit current (I_*sc*_) was measured at 2.9 µA as shown in Figure 3d. Our PVBVA MXene TENG shows better performance than other bio‐friendly electrospun TENGs.^[^ ^48^ ^]^ While PVDF‐based TENGs usually produce higher output due to fluorine's strong electronegativity,^[^ ^37^, ^49^ ^]^ our non‐fluorinated PVBVA system achieves comparable performance. This makes it a high‐performing and more sustainable alternative without the environmental issues linked to fluorinated polymers (Table S1, Supporting Information).

Figure 3e,f illustrates the triboelectric performance of the e‐tattoo when interfaced with various tribo‐materials, including polydimethylsiloxane (PDMS), nitrile, glass, aluminum (Al), and Kapton. The generated voltage amplitude is directly influenced by the difference in the electron affinities and work functions of the contacting materials; a larger difference promotes more significant charge separation and thus higher contact electrification. Materials such as nitrile, Kapton, and aluminum exhibit moderate electron‐releasing capabilities, rendering them less tribo‐positive compared to human skin^[^ ^50^ ^]^ (Figure S12, Supporting Information). These materials engage in poorer contact electrification with the e‐tattoo. The observed predominantly positive voltage outputs when TENG comes into contact with materials such as aluminum, nitrile, Kapton, and glass can be attributed to the strong electron‐accepting nature of the PVBVA matrix, which consistently draws electrons from these relatively tribopositive materials, resulting in net positive signal generation during contact‐separation cycles. PDMS generally possesses a low electron affinity, indicating a reduced tendency to acquire electrons and often acts as an electron donor in triboelectric pairings. When the e‐tattoo interfaces with PDMS, electrons are transferred from the PDMS to the strong electron‐accepting PVBVA matrix. This leads to a substantial net negative charge accumulation on the PVBVA surface. The resulting voltage peak appears as a reverse signal. The e‐tattoo achieves its highest triboelectric performance in direct contact with skin. This result is significant because conventional TENGs often require additional tribo‐materials, such as nitrile, plastics, or metals, to enhance performance. The compatibility of e‐tattoo with skin eliminates the need for such materials, simplifying the system and enhancing its suitability for wearable technology. This design leverages natural body movements for efficient energy harvesting, highlighting its potential for integration into personal electronics. The mechanical robustness of the e‐tattoo‐based TENG was evaluated through endurance testing over 3000 actuation cycles using a human finger. The TENG's electrical output remained consistent throughout the testing period. Minor fluctuations in voltage peak values in the response curve were attributed to variations in finger contact quality and applied pressure, as illustrated in Figure 3g.

**Figure 4:**
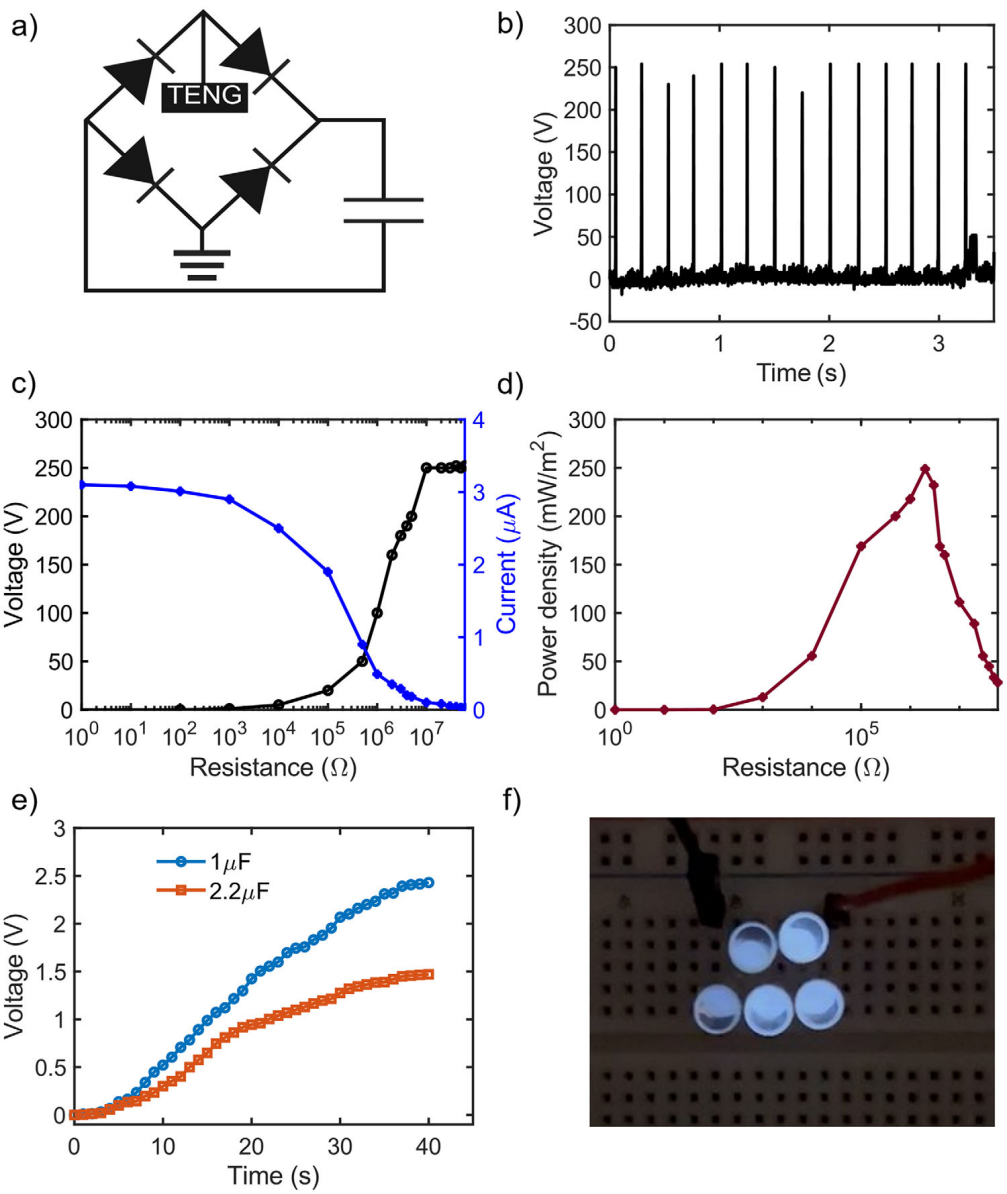
Real‐life application of the fabricated TENG. a) Schematic representation of the rectifier circuit b) Rectified voltage output of the TENG, showing the conversion of alternating current (AC) to direct current (DC) for practical applications c) Variation of voltage and current outputs with increasing load resistance, analyzing the TENG's electrical characteristics under different resistive loads d) Power density calculation of the TENG, determining the amount of power generated per unit area e) Charging curve of a commercial capacitor using the TENG activated by human skin tapping, illustrating the device's capability to store energy f) Activation of a light‐emitting diode (LED) using a rectifier circuit powered by the TENG through skin contact, demonstrating a practical application of the generated energy.

To utilize the energy harvested by the e‐tattoo, a rectification circuit was integrated to convert the alternating current (AC) output into a direct current (DC), suitable for low‐power electronic devices, as depicted in **Figure** **4**a. The performance of the TENG post‐rectification is illustrated in Figure 4b. The experimental procedure involved incrementally increasing the load resistance in the circuit while measuring the resultant voltage and current across the resistance (Figure 4c). The results revealed a clear trend as the resistance increases, the open circuit voltage (*V_oc_*) increases, while the short circuit current (I*_sc_*) decreases. The power density (*P*) was calculated using the following Equation (3):

(3) $$P=\frac{V_{\mathrm{oc}}\times I_{\mathrm{sc}}}{A}$$

where *V_oc_* is the output open circuit voltage, *I_sc_* is the short circuit current measured across the variable resistances (*R*), and *A* represents the effective area of the TENG. The peak power density achieved was ≈250 mW m^−2^ (25 µW cm^−2^) at a load resistance of 2 MΩ (Figure 4d), which is higher than reported ultrathin nanofiber‐based TENGs.^[^ ^9^, ^38^ ^]^ To demonstrate its practical applicability, the TENG was used to charge commercial capacitors via the rectification circuit, highlighting its potential for energy harvesting applications. Capacitors with capacitances of 1 and 2.2 µF were charged by manually tapping of the TENG for 40 seconds. The maximum voltage achieved was 2.45 V for the 1 µF capacitor, and 1.46 V for the 2.2 µF capacitor. These voltage levels are sufficient for powering low‐consumption electronic devices, as depicted in Figure 4e. To further illustrate the TENG's utility, we conducted a demonstration where mechanical energy, derived from finger tapping, was converted into electrical energy to illuminate a series of five light‐emitting diodes (LEDs) (Figure 4f). A real‐time illumination of LEDs was captured in Movie S2 (Supporting Information), emphasizing the device's ability. This experiment validates the potential of e‐tattoo TENG as a sustainable energy source for small electronic devices, showcasing its utility in wearable technologies and portable devices.

**Figure 5:**
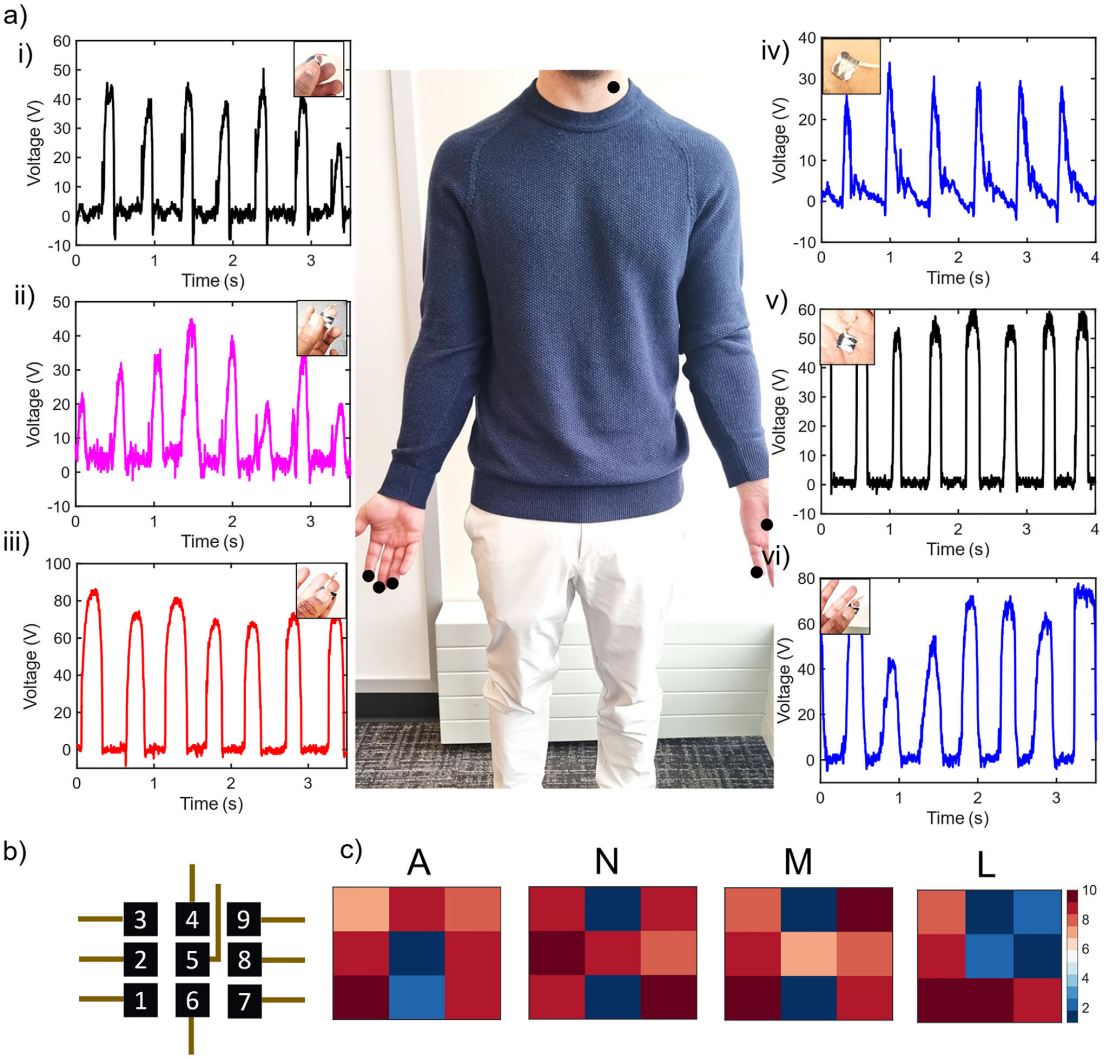
Application of the TENG as a sensor. a) Output performance of the TENG when attached to different locations on the human body: (i) index finger, (ii) middle finger, (iii) ring finger, (iv) neck, (v) palm center, and (vi) little. b) Schematic of the TENG matrix c) Output voltage and corresponding voltage pixel maps of the touch sensors as a finger traces the letters “A”, “N”, “M”, and “L” on the array. Each sensor in the array measures 1 × 1 cm.

The e‐tattoo TENG system enables real‐time monitoring of human body movements by leveraging the multisensory characteristics of the human body, which produce distinct signals during mechanical movements of various body parts. **Figure** **5**a illustrates the real‐time voltage outputs from TENGs applied to different body locations, including the palm and individual fingers. The observed voltage variations are influenced by the effective contact area and the magnitude of applied force during movement (Figure 5a i‐vi). Additionally, the e‐tattoo TENG was applied to the human neck, where its ability to record movements is demonstrated in Figure 5a‐iv and Movie S3 (Supporting Information). This capability highlights the potential of e‐tattoo TENG systems in capturing and analyzing physical motion through non‐invasive, wearable technology. To further explore its utility in human‐machine interfaces (HMIs)^[^ ^51^ ^]^ systems, the electronic e‐tattoo was configured into a 3 × 3 matrix array and attached to the human arm (Figure S13, Supporting Information). Each tattoo in the array was numbered from 1 to 9 as shown in the schematic and was mapped accordingly (Figure 5b). Upon swiping a finger across the array in a designated manner, the resulting output voltage is captured using an oscilloscope. Figure 5c displays the *V_out_* from the e‐tattoo sensor array, depicted as 3 × 3‐pixel grid, which regenerates the letters “A,” “N,” “M,” and “L.” as the finger traces these patterns, the specific voltage values corresponding to each pixel are detailed in Figure S13 (Supporting Information). This voltage mapping distinctly illustrates these letters, affirming the e‐tattoo's efficacy as a sensor element for HMIs and robotic controls. This application underscores the e‐tattoo's capability to translate human gestures into electronic outputs, providing a robust platform for advanced control mechanisms in technological applications, including gesture‐based HMIs and robotics.

**Figure 6:**
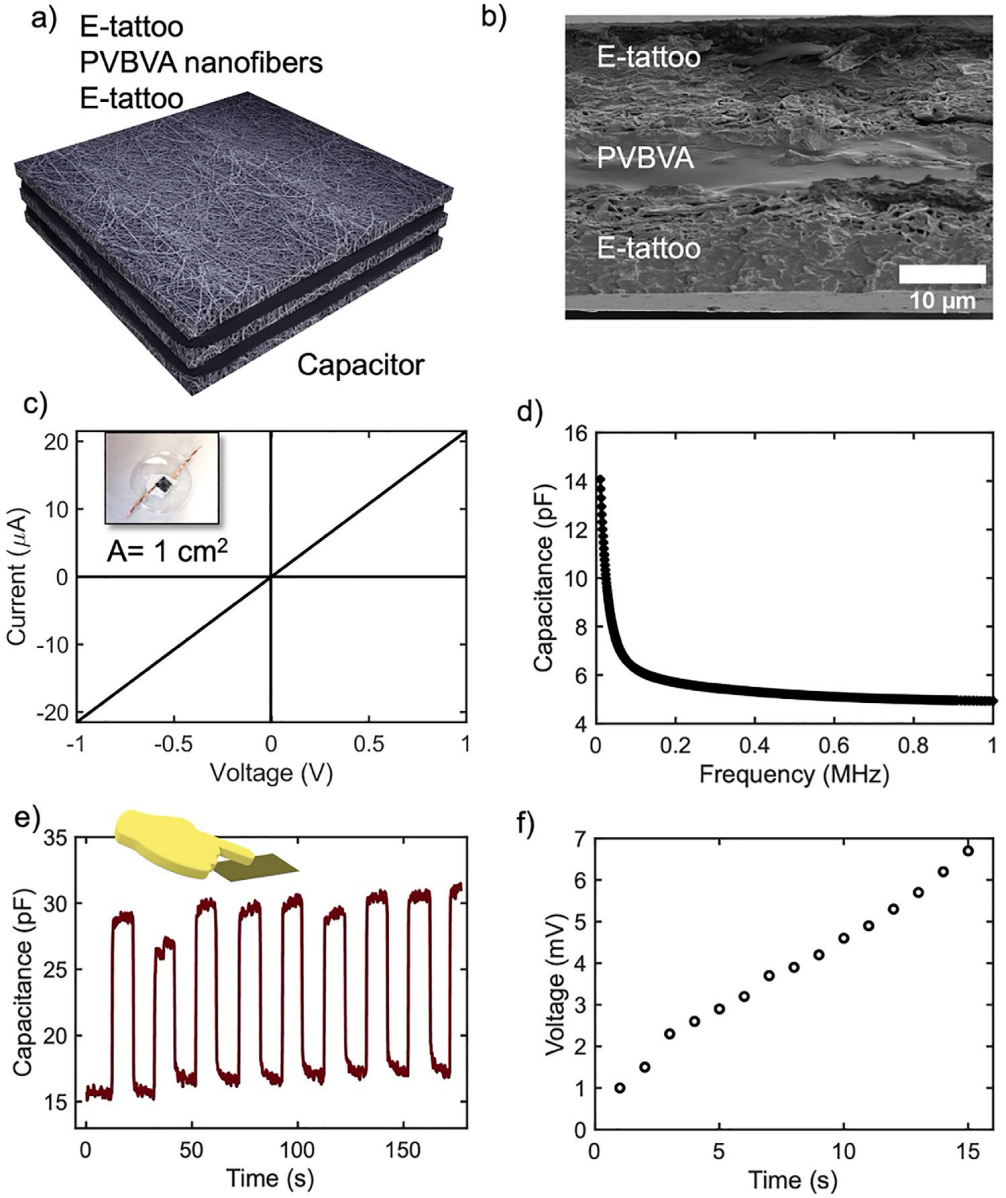
Capacitor fabrication and performance. a) Schematic illustration of the capacitor structure, highlighting the PVBVA dielectric layer. b) Cross‐sectional image of the capacitor, detailing the layered configuration. c) Current‐voltage (*I–V*) characteristics of the capacitor's conductive electrodes, demonstrating electrical behavior (Inset, the real image of the capacitor). d) Capacitance as a function of frequency at a 5 V bias, showing frequency response e). Touch‐sensitive response of the capacitor, indicating sensitivity to tactile inputs. f) Capacitor charging performance when powered by the TENG activated by a human finger, illustrating energy harvesting capability.

The constructed e‐tattoo demonstrated favorable performance in TENG applications and was subsequently employed in the fabrication of a capacitor for real‐time charge storage applications. The capacitor utilized PVBVA fiber film as the dielectric layers, while the e‐tattoo served as a conductive electrode, forming a parallel plate capacitor configuration, as depicted in **Figure** **6**a. Copper tape was employed as an external electrode for the capacitor's measurements. Other materials with higher conductivity can be used; copper was chosen due to its balance of conductivity and easy accessibility, as shown in Figure 6c **inset**, which displays an image of the fabricated capacitor. The thickness of the capacitor was ≈40 µm. SEM analysis confirmed the successful integration of multi‐layered Ti_3_C_2_T*_x_* MXene with the dielectric layer, enhancing the capacitor's charge storage performance (Figure 6b). The conductivity of the MXene electrode was evaluated through resistance measurement, with the I‐V curve (Figure 6c) demonstrating a linear relationship, indicating the good conductivity for this application. The capacitance characteristics were evaluated using a semiconductor parameter analyzer. As expected, capacitance decreases with increasing frequency, consistent with theoretical predictions. This phenomenon arises from the limited ion mobility within the dielectric layer to keep pace with the high‐frequency oscillations of the electric field, as depicted in Figure 6d. The stability of the capacitor was studied over time, and the capacitor maintained a consistent capacitance of ≈14 pF at 10 kHz under a fixed bias voltage of 5 V, as illustrated in Figure S14 (Supporting Information). The dielectric loss tangent (tan δ) further elucidates the energy dissipation characteristics of the dielectric material under an alternating electric field. Defined by the relation^[^ ^52^, ^53^ ^]^

(4) tanδ=2∗pi∗f∗R∗C

where f is frequency, R is series resistance, and C is capacitance, this parameter quantifies the material's ability to store versus dissipate energy. In the present system, the dielectric layer is composed of electrospun PVBVA fibers, while Ti_3_C_2_T_*x*_ MXene serves as the conductive electrode, as we have explained earlier. The measured tan δ reflects the intrinsic dielectric behavior of the PVBVA fiber matrix, including contributions from dipolar relaxation and charge trapping effects. A lower tan δ indicates efficient energy storage with minimal losses, while a higher value may be associated with interfacial polarization or leakage pathways in the porous fiber network. The frequency‐dependent profile of tan δ offers further insight into the dielectric relaxation dynamics (Figure S15, Supporting Information), and the observed mechanical and temporal stability of the response reinforces the material's potential for flexible and wearable energy devices. A detailed investigation of fiber‐based dielectric networks was previously conducted by Sun et al.,^[^ ^54^ ^]^ where PVDF fibers were employed to fabricate capacitive devices; our system exhibits a comparable dielectric loss behavior. Similar to their findings, we observe a frequency‐dependent decrease in capacitance and a consistent dielectric loss response, indicating that the electrospun PVBVA fiber network exhibits similar charge storage and dissipation characteristics under alternating electric fields.

For energy storage, the capacitor was tested for its functionality as a touch sensor. As shown in Figure 6e, physical contact with the capacitor led to a change in the dielectric layer thickness, increasing the capacitance from 15 pF to 30 pF. This response demonstrates the sensor's capability for capacitive sensing. While the capacitance did not fully recover after touching, likely due to the porous structure of the PVBVA fibers, the sensor demonstrated a quick response, with a rise time of 353 ms and a recovery time of 342 ms (Figure S16, Supporting Information). These values were calculated based on the standard CMOS circuit methodology, measuring the signal transition from 10% to 90% of the full response.^[^ ^55^ ^]^ This fast and stable performance confirms the sensor's suitability for touch‐sensing applications.^[^ ^40^ ^]^ To further explore its energy‐storage potential, the e‐tattoo was integrated with a rectifier circuit to charge the fabricated capacitor (Figure 4a). Energy harvesting was initiated by tapping the e‐tattoo TENG with a human finger, transferring energy and storing charge in the capacitor. The capacitor saturated quickly, achieving a maximum voltage of 6.8 mV (Figure 6f). This achieved voltage is relatively low for many applications; increasing the capacitor's surface area could significantly enhance its charge storage capacity and output voltage. By expanding the capacitor's effective area within the e‐tattoo TENG system, its energy storage capability could be improved, enabling it to store more charge and deliver higher voltages. This adjustment could significantly improve the energy harvesting capability of the e‐tattoo, making it viable for powering larger devices or extending the duration of its operation in various applications. We also tried to integrate our system to make a multi‐sensor with the same device. We used the capacitor for both capacitive touch sensor and TENG measurements. We confirmed the multimodal capability through sequential measurements, first measuring the TENG response via an oscilloscope, second, connecting the device to a Keithley 4200A to measure capacitance and touch sensitivity, and finally reattaching the oscilloscope to confirm the TENG response again. This confirms that we have successfully created a multimode sensor that can function as both a TENG and a capacitor (Movie S4, Supporting Information).

**Figure 7:**
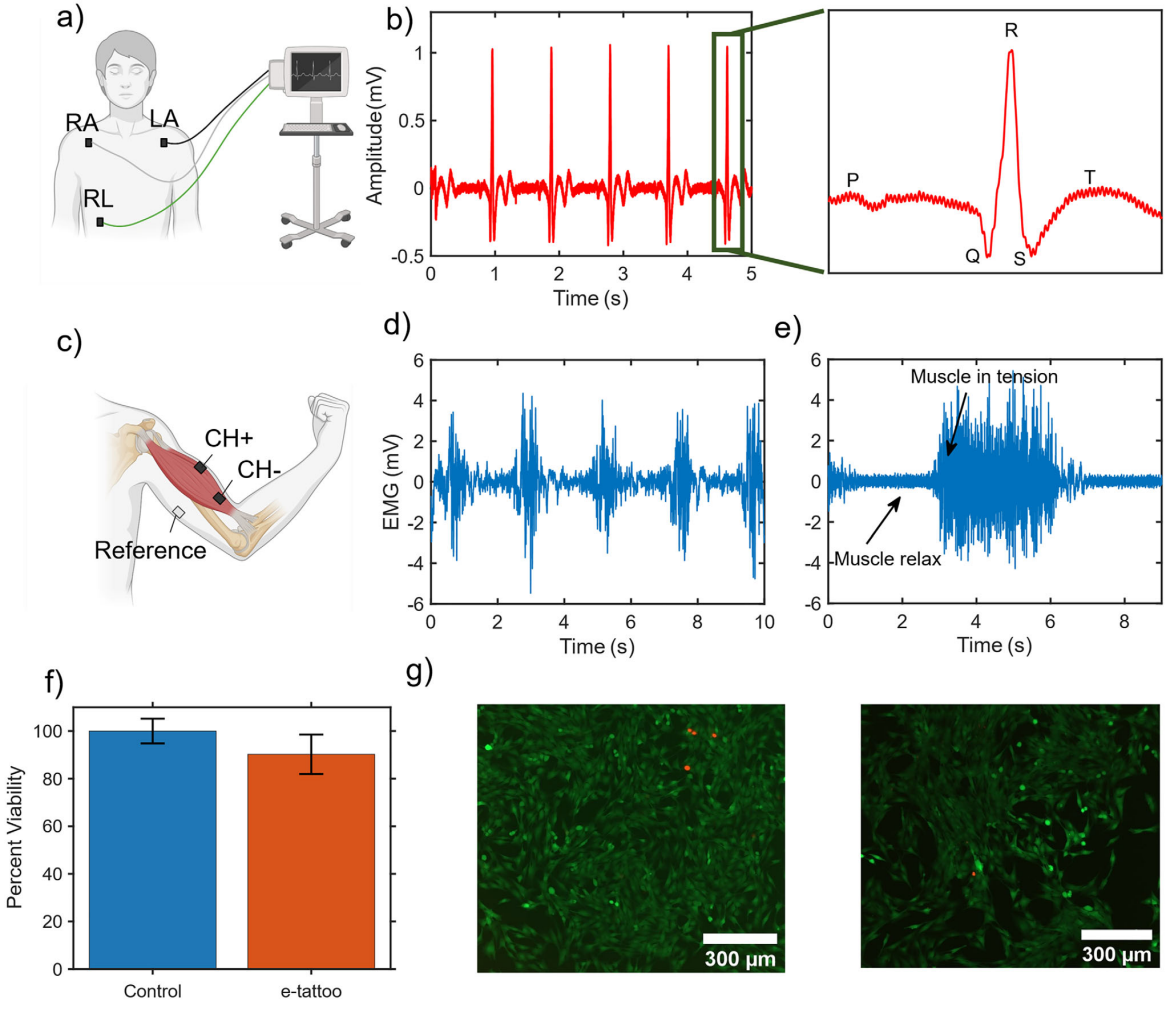
Health sensing measurements. a) Schematic of electrocardiogram (ECG) measurement setup, illustrating electrode placement at three standard locations on the human body. b) Recorded ECG signals displaying distinct PQRST peaks, corresponding to the cardiac cycle phases. c) Schematic of electromyogram (EMG) measurement setup, showing electrode placement for muscle activity detection. d) Captured EMG signals indicating muscle activity over time. e) Highlighted segment of the EMG signal demonstrating sustained muscle tension over a few‐second interval. f) Percent viability of cells in the control environment and on e‐tattoo (error bar represents the SD of 4 samples for control and 16 for e‐tattoo). g) Fluorescence image of the cells with control environment (left) and with e‐tattoo‐exposed media (right).

We further explored the potential application of the fabricated e‐tattoo for human health monitoring. A custom‐designed printed circuit board (PCB), as shown in Figure S17 (Supporting Information), incorporates an instrumentation amplifier, a high‐pass filter, an antialiasing filter, and a gain stage buffer to accurately record, filter, and amplify ECG and EMG biological signals while effectively reducing noise interference. At the core of the design is an instrumentation amplifier, chosen for its high input impedance, low offset voltage, and exceptional common‐mode rejection ratio (CMRR), which are critical for isolating weak biological signals from external electrical noise. This amplifier stage ensures signal integrity, enabling the capture of low‐amplitude ECG and EMG signals with minimal distortion and high fidelity. The ECG setup uses three electrodes to measure the heart's electrical signals, as shown in **Figure** **7**a. Their placement is carefully planned to pick up the heart's activity clearly while keeping extra noise low. Two electrodes do the main work: one, called the positive electrode, sits on the left side of the chest to catch the heart's electrical pulses, and the other, the negative electrode, goes on the right side of the chest to compare the signals. The third electrode, the reference or ground, is placed somewhere neutral, like the right leg or a bony spot where it won't pick up much heart signal. This reference keeps things steady and cuts down on background noise, like interference from nearby electronics. By looking at the difference between the left and right electrodes, the setup captures the heart's electrical changes with each beat. The recorded ECG signals clearly displayed the PQRST waveform (Figure 7b), which is crucial for clinical diagnostics. This result highlights the e‐tattoo's ability to capture accurate and reliable cardiac activity without the use of conductive gels or bulky adhesive electrodes, reinforcing its suitability for comfortable, non‐invasive health monitoring applications. For EMG measurement, the electrode configuration is optimized to enhance signal quality and suppress noise, as depicted in Figure 7c. The setup employs three electrodes: two active electrodes forming a differential pair and a third serving as a reference (ground) electrode, which is the same as ECG. The active electrodes capture the electrical activity of the target muscle (biceps), while the reference electrode establishes a stable baseline to mitigate common‐mode noise. The first active electrode, designated the recording electrode, is positioned over the muscle belly the region of maximum muscle thickness to maximize signal amplitude by directly detecting EMG signals produced during muscle contractions. The second active electrode, termed the differential electrode, is placed a few centimeters distally along the muscle fiber axis, optimizing the differential signal by minimizing spatial interference and enhancing the voltage gradient between the two points. The reference electrode is situated on an electrically inactive site as illustrated in Figure 7c, to avoid contamination from muscle potentials and provide a consistent reference voltage. This placement ensures effective cancellation of common‐mode noise, including ambient electrical interference, thereby improving EMG signal clarity. EMG recordings were obtained using three e‐tattoos affixed to the biceps, with corresponding EMG responses during muscle flexion presented in Figure 7d. In addition, Figure 7e demonstrates the tattoo's sensitivity to muscle activity. When the muscle is tightly flexed and held for few seconds and then relaxed, the e‐tattoo accurately captured the corresponding variations in signal intensity and duration.

To verify the biocompatibility of our fabricated e‐tattoo and ensure its safety for use as a wearable health sensor and energy harvester, a comprehensive cytotoxicity test was conducted. Mouse myoblast cells (C2C12, ATCC) were selected for this evaluation. The cells were seeded at a density of 5 × 10^3^ cells cm^−^^2^ in a 24‐well tissue culture plate and maintained under standard conditions for 24 hours (Experimental Section). Extraction media was prepared by soaking the e‐tattoo in standard growth media for 72 hours. Afterward, the growth medium was replaced with the extraction medium and incubated for another 24 hours. According to the Alamar blue metabolic assay, the C2C12 cells exposed to the extract exhibited a viability of 90.2% compared to 100% for the control group (Figure 7f). The minimal standard deviation indicated no significant difference between the two groups, and the live/dead cell imaging further confirmed these findings (Figure 7g). Furthermore, to gain insight into the long‐term cycling and oxidation stability of our MXene‐PVBVA composites, we performed cyclic bend testing and relative humidity testing (Figures S18 and S19, Supporting Information). The e‐tattoo was subjected to 10 000 bending cycles using an Arduino‐operated bending system, while the resistance was measured every 1000 cycles and found that the total resistance change after 10 000 cycles was ≈35%, showing good cyclic stability for a wearable system. Humidity had a larger effect, where resistance increased to 80% over 10 days in an increasing relative humidity environment from 30% – 50%. These stability results suggest that advanced packing methods to improve bendability and oxidation resistance may further improve our e‐tattoos' performance.

## 3 Conclusion

This study introduces a multifunctional, skin‐compatible e‐tattoo that integrates energy harvesting, charge storage, and health monitoring into a single, ultrathin platform. Fabricated with PVBVA fibers functionalized with Ti_3_C_2_T*_x_* MXene, the e‐tattoo exhibits excellent mechanical stability, conformability, and electrical conductivity. The integrated triboelectric nanogenerator (TENG) efficiently harvests energy from body movements, while the capacitor demonstrates reliable charge storage with touch sensitivity. The e‐tattoo successfully records real‐time electrophysiological signals, including ECG and EMG, emphasizing its potential for continuous health monitoring applications. The demonstrated 3×3 touch sensor matrix further illustrates the e‐tattoo's suitability for human‐machine interfaces. This work advances flexible electronics by offering a robust, multifunctional solution for wearable technologies. Its versatile features position it as a promising candidate for applications in energy management, biomedical monitoring, and interactive systems, paving the way for next‐generation wearable devices.

## 4 Experimental Section

### Preparation of PVBVA Fibers

First, a solution of PVBVA (Mw 50,000–80,000, Sigma–Aldrich, CAS Number: 27360‐07‐2) in ethanol was prepared for the electrospinning process. In this instance, a concentration of 5 wt.% PVBVA in ethanol was used. The prepared solution was loaded into a syringe (18 G) and attached to the electrospinning apparatus. A high voltage of 15 kV was applied with a distance between the nozzle and the collector set at 20 cm. After 30 minutes of electrospinning, the desired fibers were obtained.

### Multilayered Ti_3_C_2_T_x_ MXene Synthesis

Ti_3_AlC_2_ MAX phase (>98 wt.%, 400 mesh) was purchased from NANOPLEXUS. For the synthesis of multilayered (*ml‐)* Ti_3_C_2_T*_x_* MXene, a minimally intensive delamination (MILD) methodology was used, as previously detailed.^[^ ^56^ ^]^ First, an etchant was prepared by mixing 10 mL of hydrochloric acid (36.5 to 38.0%, Fisher Chemical TM) and 3.2 g of lithium fluoride (98.5%, ‐325 mesh powder, Alfa Aesar) in a high‐density polyethylene container. The synthesized Ti_3_AlC_2_ MAX phase powder (2 g) was gradually added to the etchant over 10 min and stirred at 500 rpm for 72 h at 50 °C. The mixture was subsequently washed via centrifugation at 3500 rpm for 5 mins with ultrapure water until the pH of the supernatant reached to 5–6. Finally, the (*ml‐)* Ti_3_C_2_T*_x_*, obtained as a black slurry, was collected using a spatula and vacuum dried at room temperature overnight. To verify the successful synthesis of MXene post‐etching, XRD analysis was conducted on both the MAX phase and the resulting MXene. The disappearance of the aluminum (Al) peak at 2θ = 39° and the shift of the (002) peak from 10° to 7° confirmed the effective removal of Al and the formation of MXene, as shown in Figure S20 (Supporting Information). Additionally, SEM and EDS were performed on the MAX phase and MXene, further validating the multilayer structure of the synthesized MXene (Figure S20, Supporting Information).

### Preparation of Ti_3_AlC_2_ MXene Ink for E‐Tattoo

To prepare MXene ink suitable for brush painting, 300 mg of synthesized Ti_3_C_2_T_*x*_ MXene was combined with 2.5 mL of ethanol and 2.5 mL of ethylene glycol and homogenized using a shear mixer to ensure a uniform dispersion.

### Preparation of E‐Tattoo

After preparing the MXene ink and PVBVA fibers, the fibers were cut into the desired shape and affixed to a glass slide using Kapton tape. Subsequently, a painting brush was used to uniformly coat the fibers with a layer of MXene ink. The entire assembly was then placed in a glove box under an argon atmosphere for at least 24 h to ensure proper curing and environmental isolation. To assess the homogeneity of the MXene coating on the e‐tattoo layer, EDS was employed. EDS imaging revealed a uniform distribution of titanium (Ti) across the surface, confirming consistent MXene deposition. Trace amounts of fluorine (F) were detected, indicating the use of hydrofluoric acid (HF) during MXene etching, while the absence of aluminum (Al) confirmed complete removal of the precursor MAX phase (Figure S21, Supporting Information).

### Preparation of FTIR Samples

FTIR pellets were prepared to analyze the spectral characteristics of PVBVA and e‐tattoo (PVBVA/Ti_3_C_2_T_*x*_) composites. For the PVBVA sample, 1.5 mg of precipitated PVBVA was finely ground with 200 mg of KBr and pressed under 8 tons of pressure for 4 min to form a transparent pellet suitable for FTIR analysis. For the e‐tattoo sample, the same procedure was initially followed by grinding 1 mg of the composite with 200 mg of KBr and pressing it under identical conditions. However, due to the opacity of MXene, at this concentration the FTIR spectra showed 0% transmittance at multiple peaks. To overcome this limitation, the pellet was ground with a mortar and pestle. 50 mg of this sample was mixed with 150 mg of fresh KBr and pressed into a new pellet. The FTIR spectra were then successfully recorded in transmittance mode, ensuring an optimized signal for spectral interpretation.

### Cytotoxicity Assessment of PVBVA/MXene Composite

Mouse myoblast cells (C2C12, ATCC) were used for the cytotoxicity assay. C2C12 cells were cultured in standard growth media, Dulbecco's Modified Eagle's Medium (ATCC, 30–2002) supplemented with 10% fetal bovine serum (Fisher Scientific, A38401‐01), and penicillin and streptomycin (100 U/ml) (Gibco, 218 754). C2C12 were cultured at 37 °C and 5% CO_2_ under humid conditions. The extract test was conducted as described by ISO 10993–5. The PVBVA/MXene e‐tattoo was prepared and cut into 6.25 cm^2^ pieces and sterilized under a UV light for 10 minutes before soaking in 5 ml of growth media for 72 hours at 37 °C and 5% CO_2_ under humid conditions. The prepared media was disinfected using a sterile 0.22 µm filter. C2C12 cells were seeded at 5 × 10^3^ cells cm^−2^ in a 24‐well tissue culture plate under standard conditions for 24 h. Growth media was removed, and extraction media was applied to cells for 24 h. Alamar blue metabolic assay was completed by adding 10% (v/v) Alamar blue in growth media to the 24‐well plate and incubated for an additional 4 h. Control samples with media only and media with cells were prepared, and all samples. Fluorescence was measured using an Agilent Citation 10 plate reader using excitation/emission 560/590 nm.

### Characterization and Measurements

The morphology of the printed TENG was characterized using scanning electron microscopy (SEM, FEI Teneo FESEM). Voltage measurements were conducted using a digital storage oscilloscope (Tektronix, TBS2204B), and current measurements were performed using a Keithley electrometer (Model 6517B). All TENG measurements were carried out at a frequency of 3 Hz, applying an approximate force of 5 N. Commercial diodes were utilized to construct the rectifier circuit. For MXene washing, an Allegra 64 R centrifuge was used. FTIR spectrometers, TENSOR 27 were used for FTIR measurement. Keithley 4200A was used to measure the electrical measurements. The mechanical test frame was a Shimadzu EZ Test EX‐SZ with a 10 N load cell. Test rate was 2 mm min^−1^. Samples had a gauge length between 11–15 mm. Scanning Kelvin probe force microscopy (SKPFM) was performed using a Bruker Dimension Icon atomic force microscope (AFM) operating with PeakForce Tapping^[^ ^57^ ^]^ frequency modulation (PF FM‐KPFM, Bruker). For cell imaging, the Invitrogen Live/Dead Cell Imaging kit (ThermoFisher, R37601) was used. Fluorescence images were taken using GFP (488/515 nm) and Texas Red (570/602 nm) filter sets.

### EMG and ECG Measurements

A custom printed circuit board (PCB) was designed to amplify, filter, and record biological signals. The signal chain consisted of an instrumentation amplifier (INA350, gain = 50x), a high‐pass filter (1 Hz), and an antialiasing filter (200 Hz) before acquisition with an oscilloscope (Digilent, Analog Discovery 2). EMG and ECG recordings were fully differential, where the third electrode acted as a driven reference electrode. Data were filtered post‐acquisition in MATLAB using an acausal notch filter to reduce 60 Hz interference. Written consent from the research participant was obtained for collecting biometric data with e‐tattoos. Institution review board consent was not required due for individual case studies.

### Statistical Analysis

All data were analyzed using MATLAB 2023b. Error bars represent the standard deviation (mean ± SD) based on at least three independent samples for each experiment. Outliers were evaluated and removed where necessary to ensure data reliability. Figures were generated using PowerPoint for a publication‐quality presentation.

## Conflict of Interest

The authors declare no conflict of interest.

## Author Contributions

A.P. contributed to conceptualization, data curation, formal analysis, and writing of the original draft. F.R.K. prepared the MXene ink and assisted with draft editing. H.B. and A.R. performed SEM characterization and contributed to draft editing. H.B. conducted cytotoxicity testing. M.C. and C.M.E. carried out SKPFM operation and data analysis. M.L.N. performed mechanical analysis and contributed to draft editing. F.N.M. and B.C.J. conducted ECG and EMG measurements and assisted with draft editing. T.V.V. contributed to conceptualization and draft editing. J.E. performed FTIR analysis and contributed to draft editing. A.B. conducted mechanical measurements. B.J. and Z.D. contributed to manuscript review, editing, and drafting. K.C. performed electrospinning and assisted with draft review and editing. C.E.S. contributed to draft review and editing. D.E. contributed to conceptualization, writing, review and editing, and supervision.

## Supporting information



Supporting Information

Supplemental Movie 1

Supplemental Movie 2

Supplemental Movie 3

Supplemental Movie 4

## Data Availability

The data that support the findings of this study are available from the corresponding author upon reasonable request.
