# Supplementary material for: Multifunctional E‐Tattoos Based on Electrospun PVBVA Fibers Coated with Ti3C2T x MXene for Energy Harvesting, Energy Storage, and Biometric Sensing
Source: Adv Sci (Weinh). 2025 Dec 7;13(11):e18697. doi: 10.1002/advs.202518697 (PMC12931238; doi:10.1002/advs.202518697)
Supplement: Supplementary file 1 — Supporting Information [file ADVS-13-e18697-s005.docx]

**Supporting Information**

Multifunctional E-Tattoos Based on Electrospun PVBVA Fibers Coated with Ti_3_C_2_T*_x_* MXene for Energy Harvesting, Energy Storage, and Biometric Sensing

Ajay Pratap^1^, Fereshteh Rajabi Kouchi^1^ Tony Valayil-Varghese^1,2^, Hailey Burgoyne^1,2^, Attila Rektor^1^, Michael Curtis^1^, Miranda Lea Nelson^3^, Francis N. Mokogwu^4^, Corey M. Efaw^1,2^, Josh Eixenberger^2,5,6^, Allyssa Bateman^1,2,6^, Benjamin C. Johnson^4^, Brian Jaques^1,2,6^, Zhangxian Deng^6,7^, Kurtis Cantley^4^, Christopher E. Shuck^8^_,_ David Estrada^1,2,6,9,*^

^1^Micron School of Material Science and Engineering, Boise State University, Boise, ID, 83725, USA.

^2^Micron Center for Materials Research, Boise State University, Boise ID, 83725, USA.

^3^Biomedical Engineering Doctoral Program, Boise State University, Boise, ID 83725, USA

^4^Department of Electrical and Computer Engineering, Boise State University, Boise, ID, 83725, USA.

^5^Department of Physics, Boise State University, Boise, ID, 83725, USA.

^6^Center for Advanced Energy Studies, Boise State University, Boise ID, 83725, USA.

^7^Mechanical and Biomedical Engineering, Boise State University, Boise, ID, 83725, USA

^8^Department of Chemistry and Chemical Biology, Rutgers University, Piscataway, NJ, 08854, USA.

^9^Idaho National Laboratory, Idaho Falls, ID,83415, USA.

*Corresponding Author: [daveestrada@boisestate.edu](mailto:daveestrada@boisestate.edu)

Figure S1-S21: Supporting experimental data.

Table S1: Comparison of PVDF- MXene TENG with PVBVA based TENG

Movie M1: Removal of E-tattoo

Movie M2: LED’s glowing

Movie M3: Real time voltage output

Movie M4: Multimode capability of Device through sequential measurements


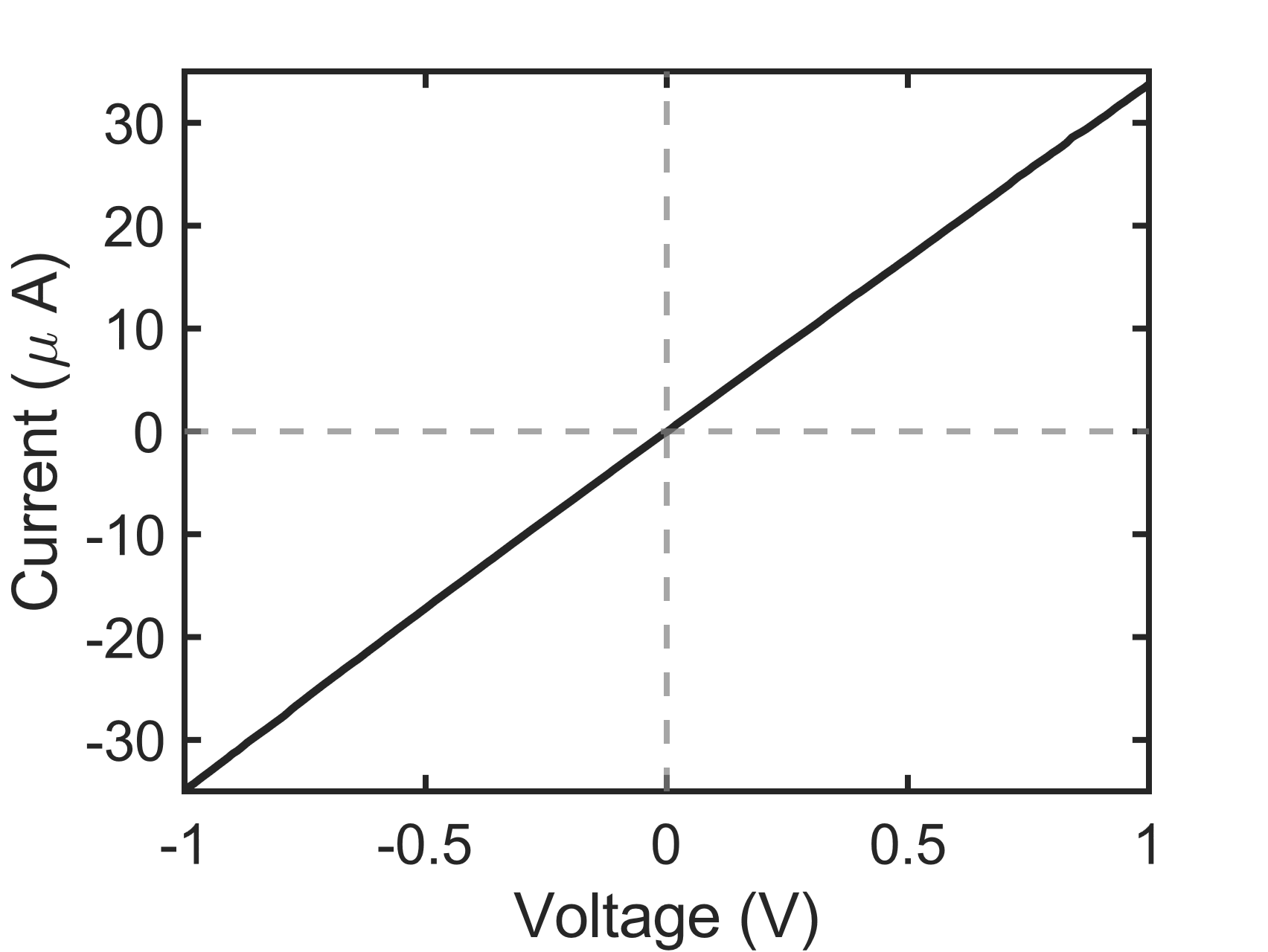


**Figure S1**: I-V plot confirms the ohmic contact and high conductivity of the prepared e-tattoo when placed on human skin. The graph displays the measured current (µA) as a function of the applied voltage (V) across the e-tattoo electrodes.


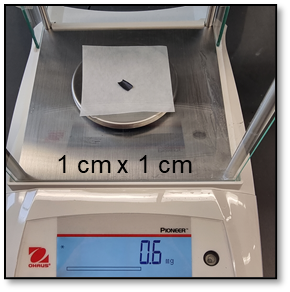


**Figure S2**: Direct measurement of the ultra-low mass of the fabricated e-tattoo. The figure shows a high-resolution digital balance displaying the mass of a 1 cm x 1 cm tattoo sample. The measured weight is 0.6 mg.


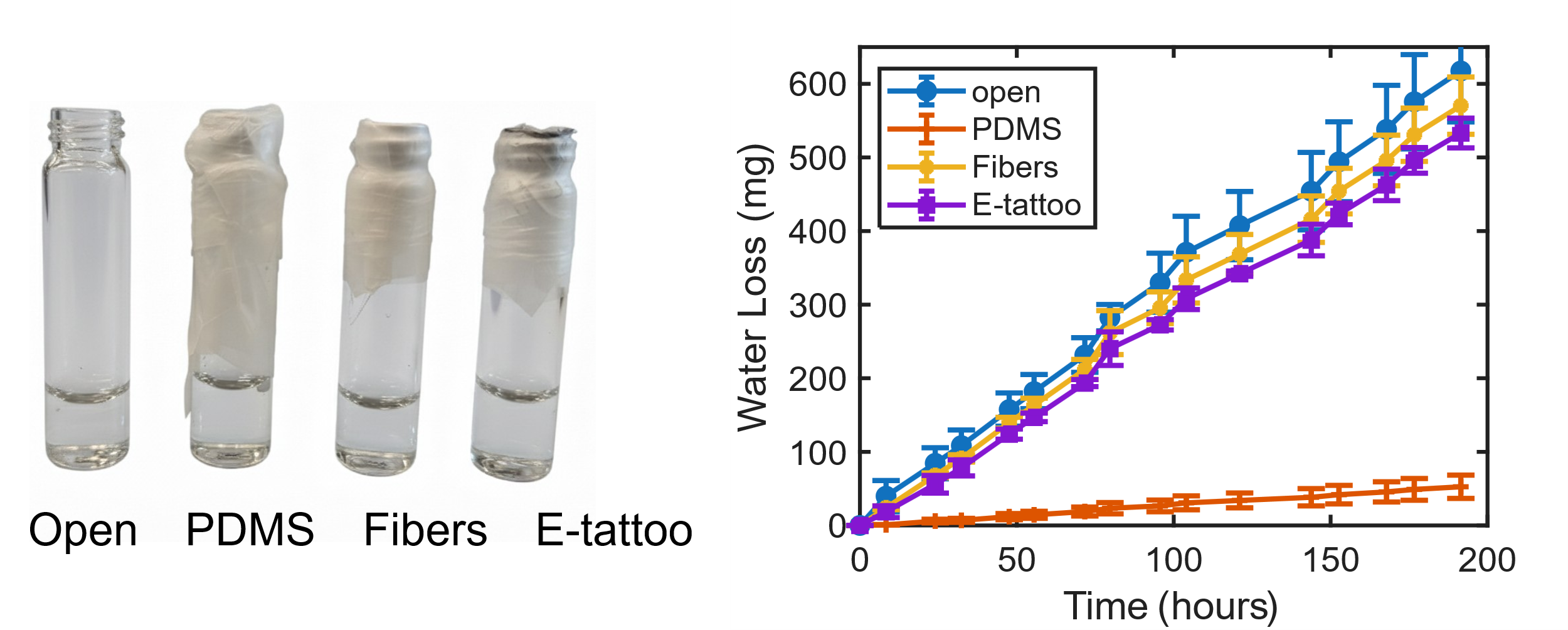


**Figure S3:** Experimental setup for Water Vapor Transmission Rate (WVTR) measurement (left) and comparison of water loss over time (right) among an open vial and vials sealed with PDMS, electrospun PVBVA fibers, and PVBVA/MXene e-tattoo films. Error bars represent the standard deviation (SD) across three samples for each condition.


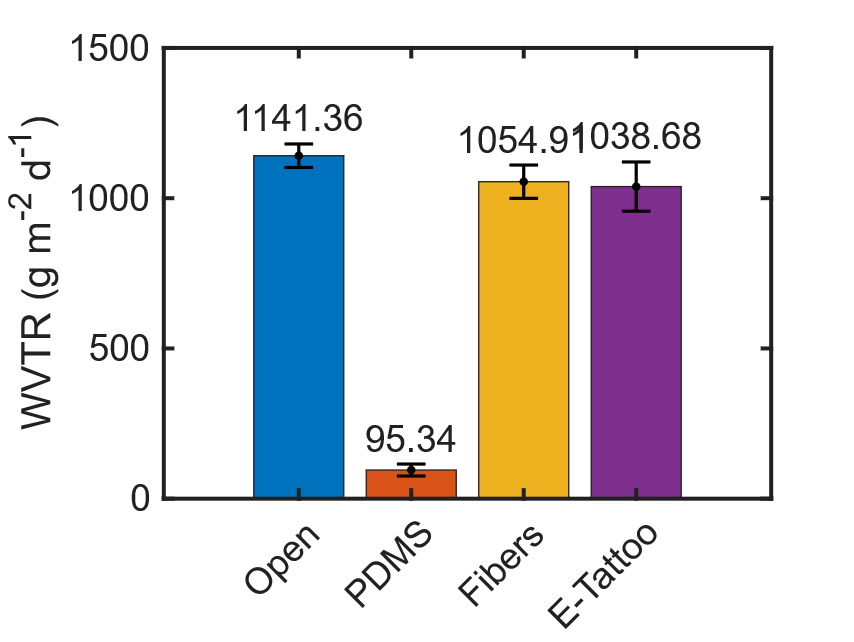


**Figure S4:** Histogram comparing the water vapor transmission rate (WVTR) among an open vial and vials sealed with PDMS, electrospun PVBVA fibers, and the PVBVA/MXene e-tattoo films, highlighting the superior breathability of the fiber-based and e-tattoo membranes compared to PDMS. Error bars represent the standard deviation (SD) across three samples for each condition.


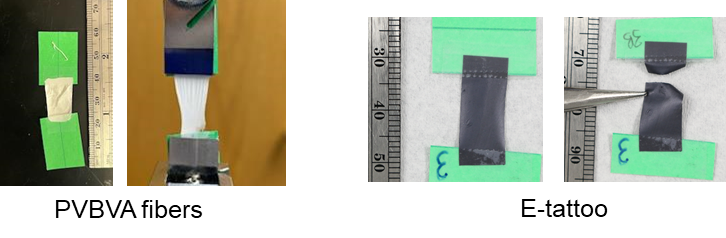


**Figure S5**: Visual demonstration of PVBVA fibers and e-tattoo mechanical durability. Images illustrating the preparation for mechanical performance of the PVBVA and e-tattoo. The two images on the left show the PVBVA nanofiber substrate, including its extreme elongation during tensile testing (center-left image). The two images on the right display the finished e-tattoo device samples before and after being subjected to significant mechanical strain.


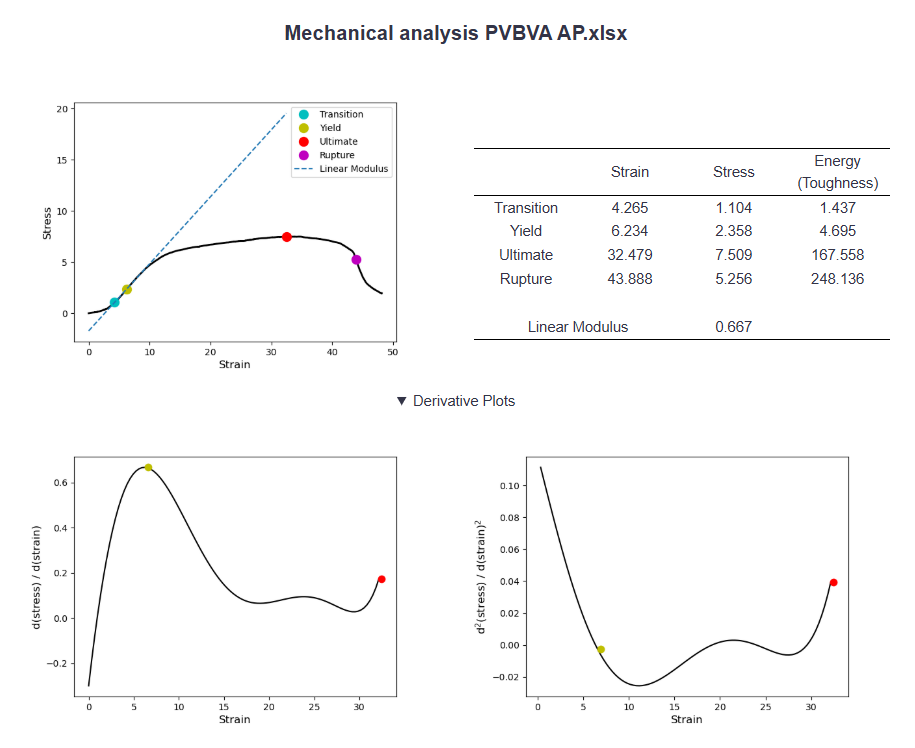


**Figure S6.** Stress-Strain analysis and mechanical parameter determination for the PVBVA nanofiber substrate. The top-left graph is the Stress-Strain curve of the PVBVA nanofiber, with key mechanical points (Transition, Yield, Ultimate, Rupture) highlighted. The Top-Right Table quantifies the corresponding Strain, Stress, and Energy (Toughness), for these points. The high ultimate stress (7.509 MPa) exceptional material resilience. The Bottom Panels show the first and second derivative plots used to precisely locate the yield and ultimate points.


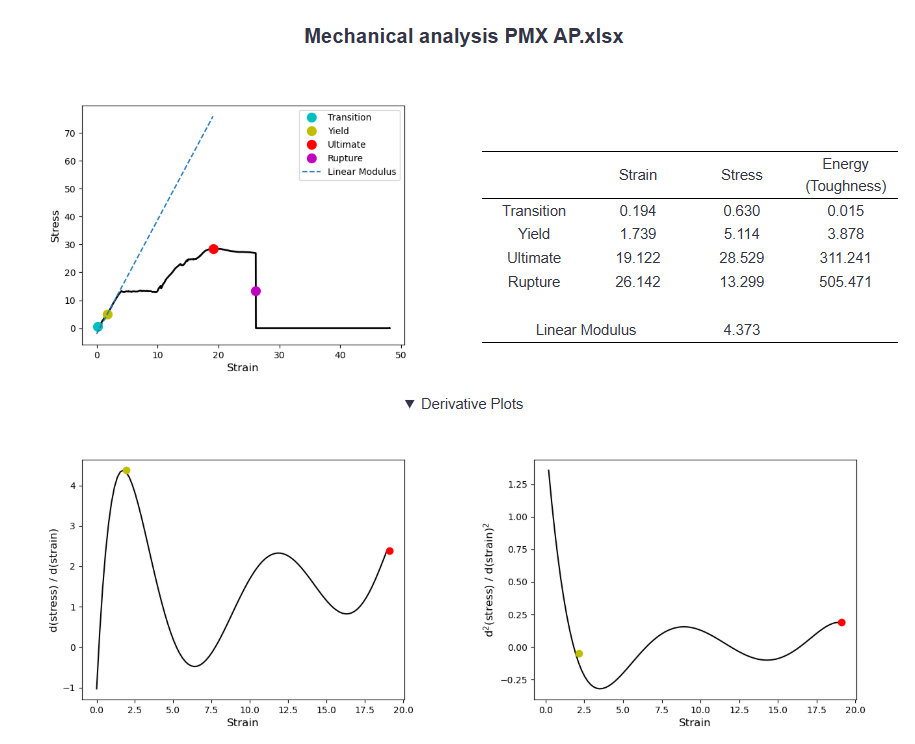


**Figure S7.** analysis and mechanical parameter determination for the e-tattoo substrate. The top-left graph is the Stress-Strain curve of the PMx, with key mechanical points (Transition, Yield, Ultimate, Rupture) highlighted. The Top-Right Table quantifies the corresponding Strain, Stress, and Energy (Toughness), for these points. The high ultimate stress (28.529 MPa) exceptional material resilience. The Bottom Panels show the first and second derivative plots used to precisely locate the yield and ultimate points.


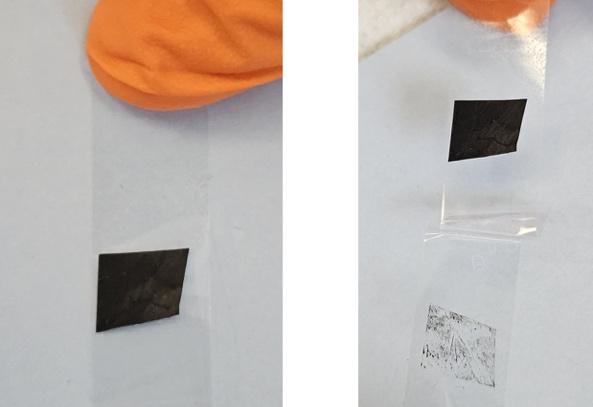


**Figure S8**: Adhesion testing of MXene layer to PVBVA conducted using a practical tape test following IPC-650-2.4.1. Two adhesive tapes were used to sandwich the e-tattoo, with one side attached to the fiber layer and the other to the MXene surface. Upon peeling the MXene-side tape, only minimal detachment of the MXene layer was observed, while no visible separation occurred between the MXene and PVBVA fibers.

**Figure S9**. Working mechanism of the single-electrode TENG. This diagram illustrates the operational principle of the single-electrode TENG, which uses the skin as the second triboelectric layer and the ground as the reference. The e-tattoo consists of a conductive Ti_3_C_2_T_x_ layer as electrode coated on the PVBVA nanofiber substrate. Contact: When the device contacts the positively-charged human skin, there is an equilibrium (no charge induced). Separation: As the device separates from the skin, the induced potential difference drives electrons from the single MXene electrode through the external resistor R to the ground. Approach: As the skin approaches the device again, the potential difference reverses, driving electrons from the ground back to the MXene electrode, completing the cycle and generating an Alternating Current (AC) output from human movement.


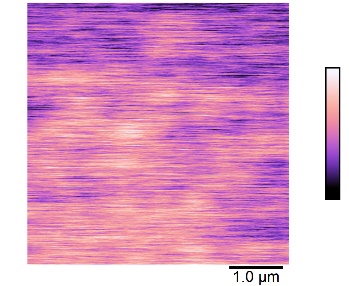

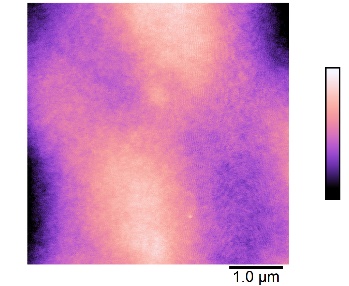


10 nm

800 mV

**Figure S10.** Surface characterization of the PVBVA layer is shown in two panels: (Left) The topography map captures nanoscale height variations with a standard deviation of 1.59 nm and a total range of 10.8 nm. (Right) The Volta potential difference map details the spatial distribution of surface potential, measured relative to Au, spanning a range of 858 mV. This uniform mapping of height and potential demonstrates the consistent morphology and triboelectric properties of the PVBVA layer, confirming its suitability for stable surface charge and sensing applications.


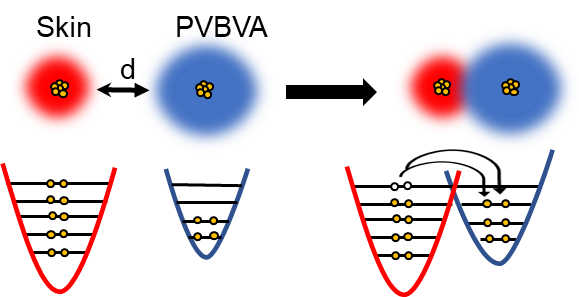


**Figure S11.** Electron cloud and potential well model for triboelectrification. This schematic illustrates the charge transfer mechanism between two materials with differing electron affinities. The left side shows the separated materials and their respective potential wells. The right side shows contact, where electrons flow from the potential well of skin to the potential well of PVBVA. This transfer results in the static charge separation needed to drive the single-electrode TENG.


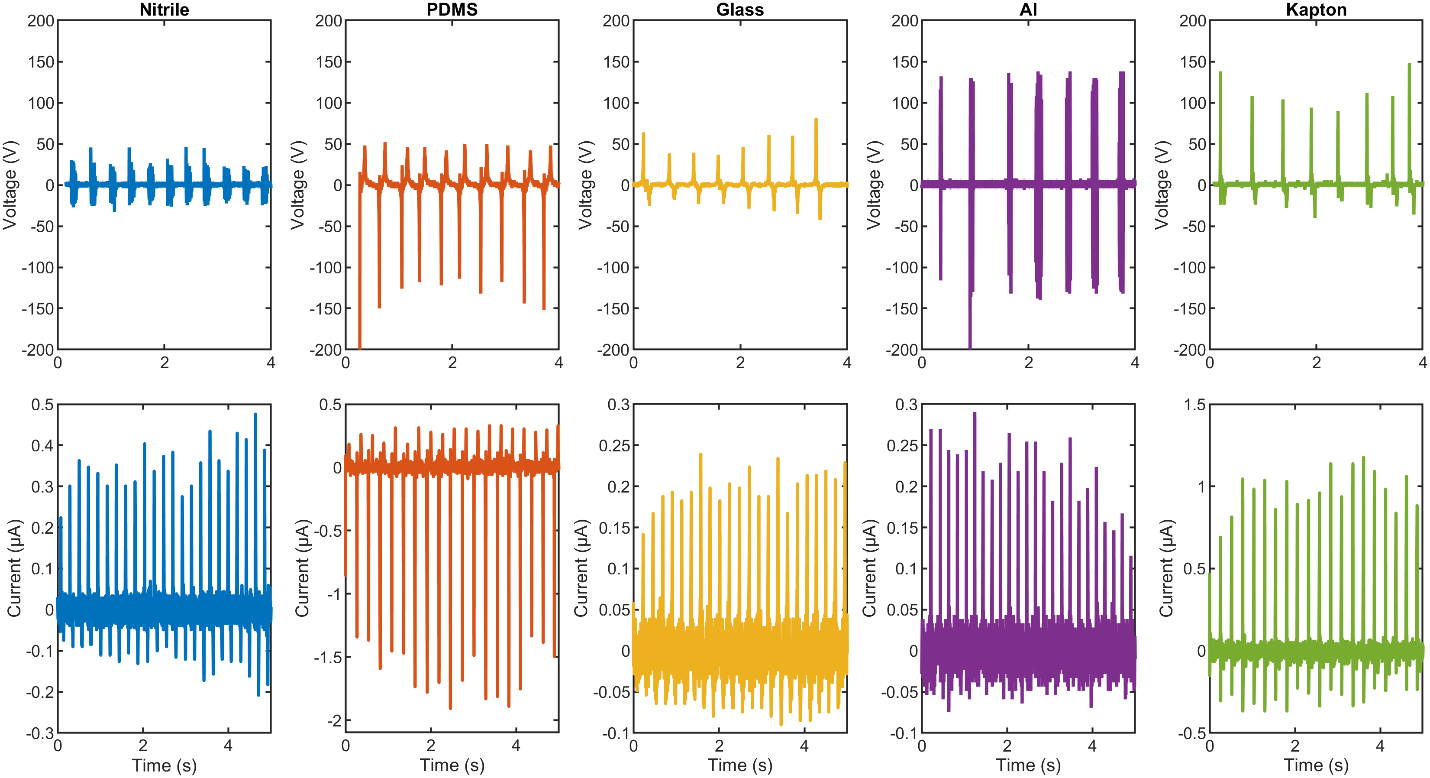


**Figure S12.** Comparative electrical output of the single-electrode TENG against various triboelectric materials. The figure presents the measured open-circuit voltage (top row) and short-circuit current (bottom wow) generated by the single-electrode TENG when contacted with five different materials: Nitrile, Polydimethylsiloxane (PDMS), Glass, Aluminum (Al), and Kapton.

**Figure S13**: The figure showcases the tactile detection abilities of the 3×3 e-tattoo matrix adhered to the forearm. The accompanying graph records voltage signals (V) from individual matrix pixels (A, N, M, and L) over time (s) as different locations are pressed or swept by touch. Each contact event triggers clear, sharply defined voltage peaks solely in the touched pixel’s data trace, while non-contacted pixels display minimal activity. This pronounced, localized electrical response underscores the matrix’s high spatial resolution and selectivity as a self-powered tactile sensor.


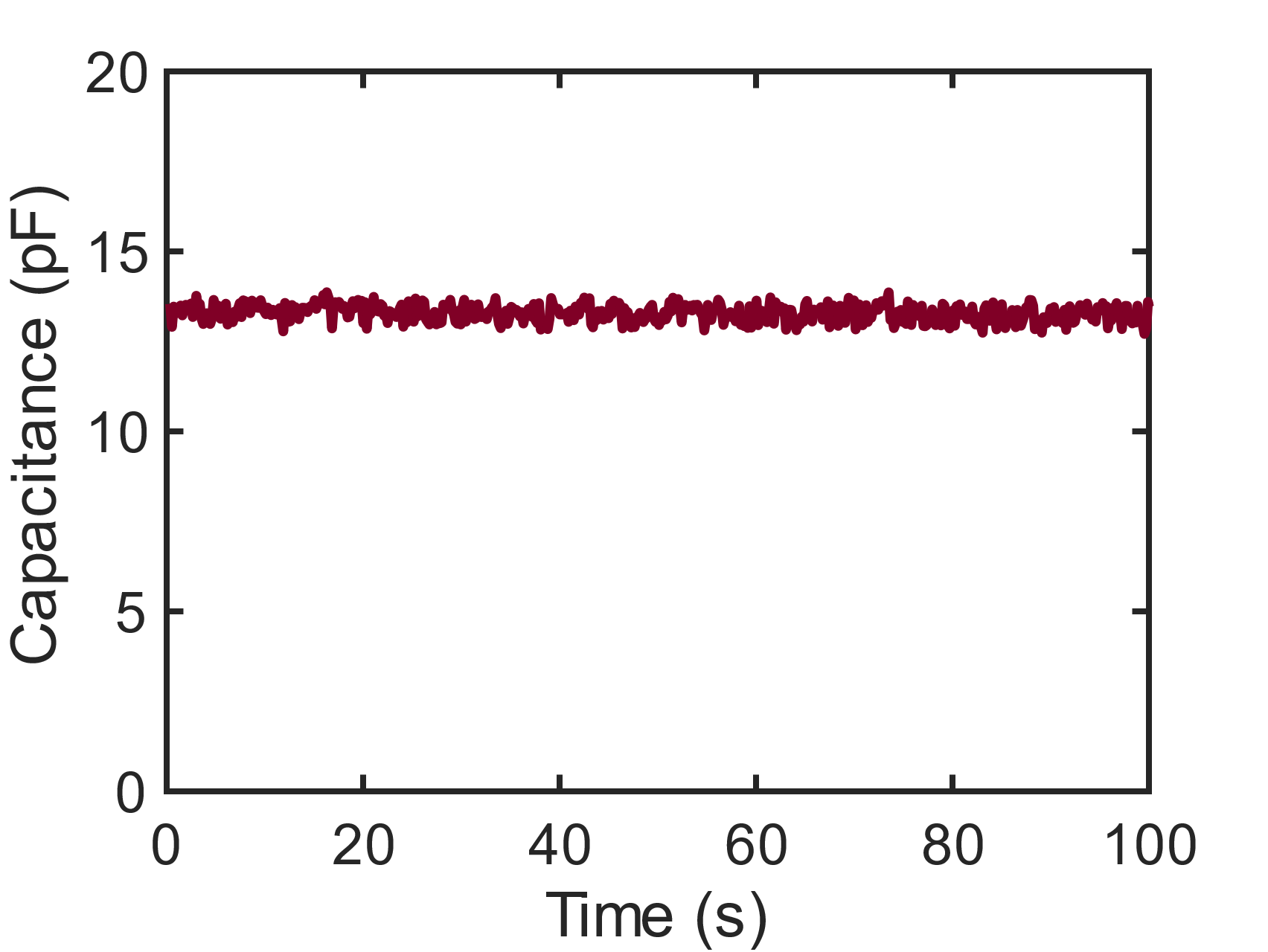


**Figure S14.** Stability of the e-tattoo capacitance over time. The plot displays the measured capacitance (pf) of the fabricated e-tattoo as a function of time (s), over a 100 s period. The measurement was conducted at a fixed frequency of 10 kHz and a constant bias voltage of 5 V. The trace shows a stable, near-constant capacitance value of approximately 14 pf with minimal fluctuation. This result confirms the excellent dielectric and electrical stability of the e-tattoo structure under continuous operation, which is critical for long-term sensing reliability.


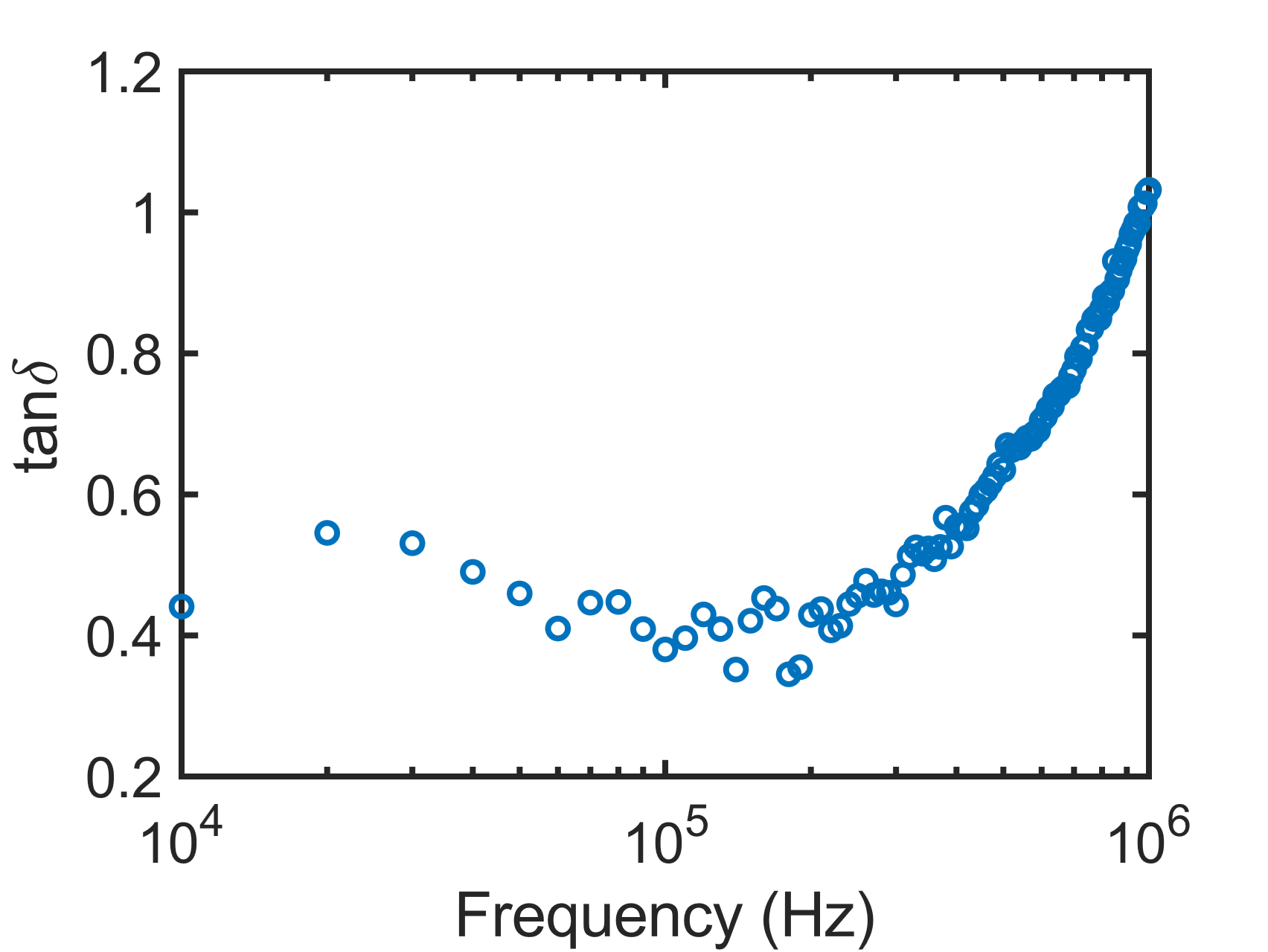


**Figure S15**: The plot displays the measured dielectric loss tangent (tan *δ*) versus frequency, ranging from 10 kHz to 1 MHz on a logarithmic scale. The initial low values and nearly flat profile up to approximately 100 kHz indicate that the device maintains stable dielectric properties within the range expected for typical operational use.


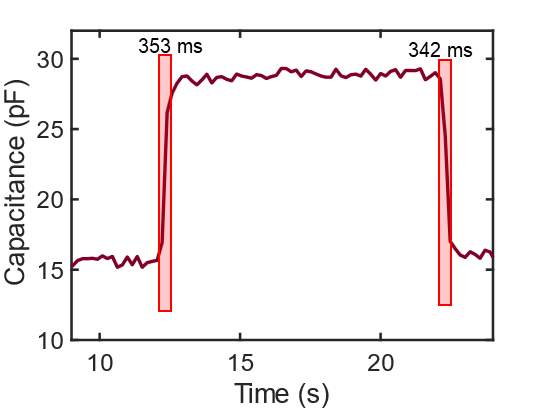


**Figure S16**: Measured response and recovery time of the capacitive touch sensor. The plot highlights the rapid transient behavior of the e-tattoo capacitive sensor following a touch. The response time, defined as the rise from baseline capacitance (∼15 pF) to its peak activated value (∼30 pF), is measured at 353 ms. Correspondingly, the recovery or decay time back to baseline is 342 ms. These sub-second timescales verify the sensor’s capability for fast, real-time tactile detection, supporting its application in dynamic, interactive wearable technologies.


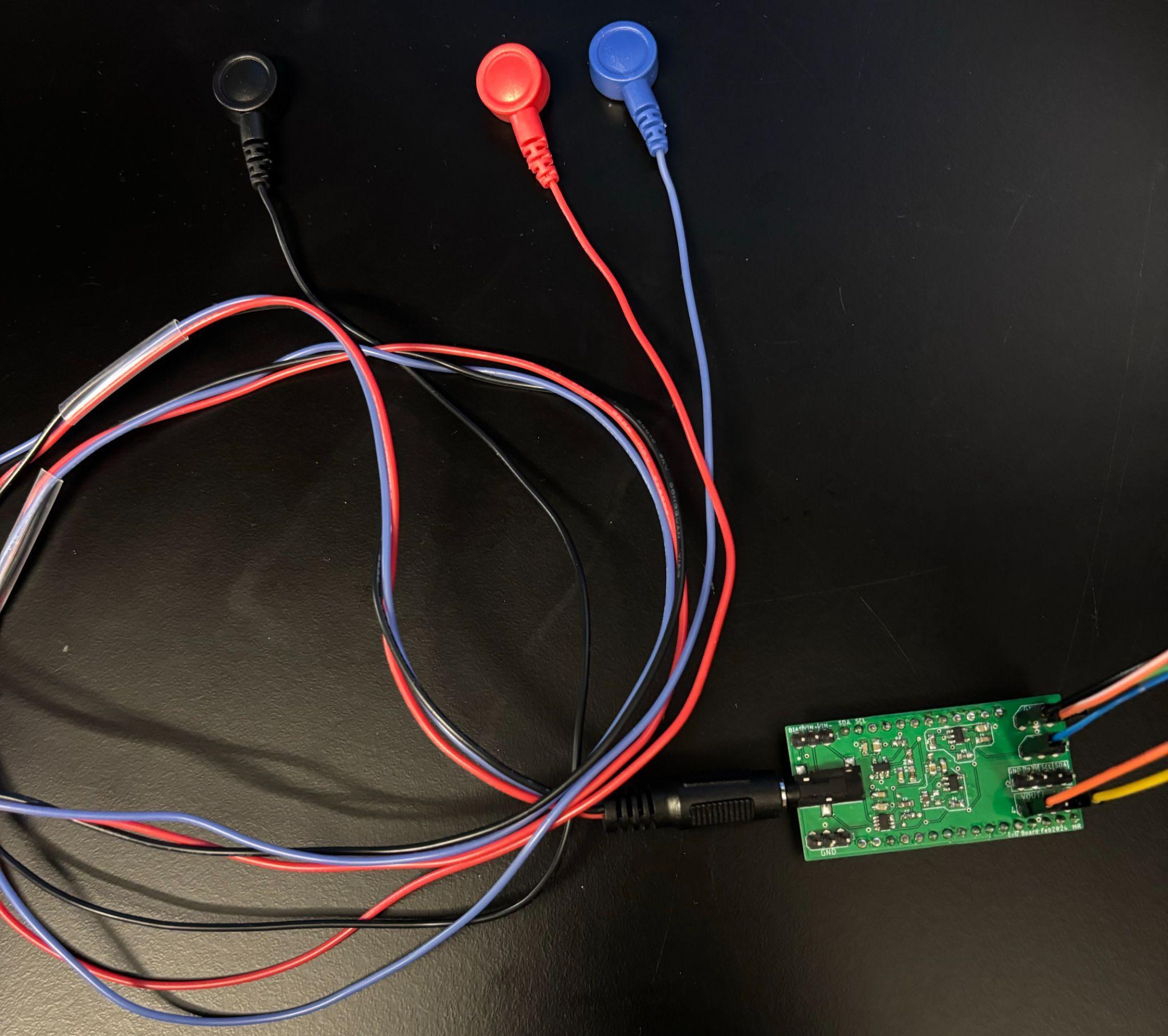


**Figure S17:** Custom-designed printed circuit board connected to three color-coded leads (black, red, and blue) for ECG and EMG signal acquisition, illustrating the experimental setup used for biopotential measurements.


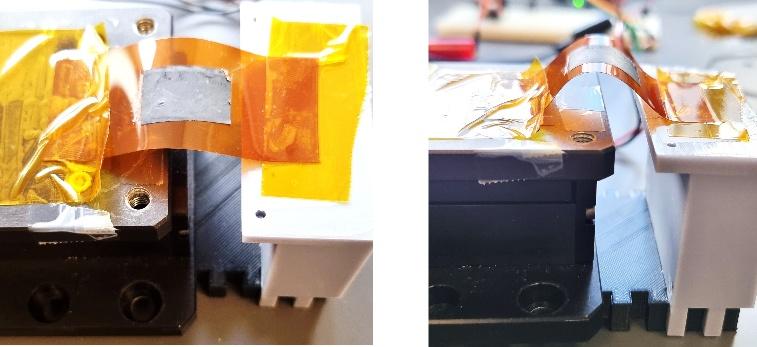


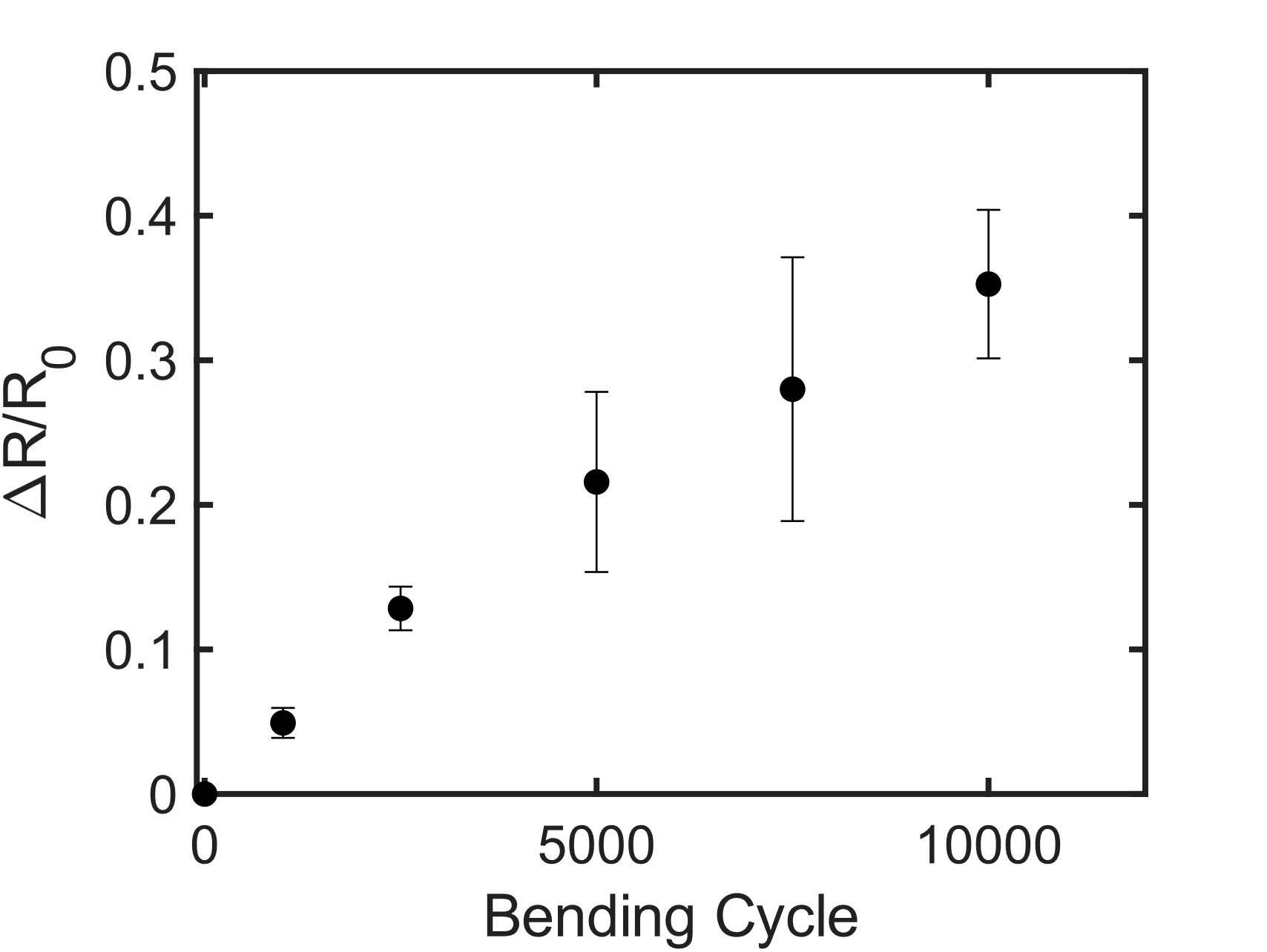


**Figure S18:** E-tattoo attached on Kapton during bending test and the resistance change of the e-tattoo as a function of the number of cyclic bending operations. The data shows resistance degradation over 10,000 bending cycles. Error bars represent the SD across three samples.

**Measurement of stability**: Three identical samples (n=3) were subjected to a progressive increase in ambient relative humidity (RH) within a chamber. The measurement cycle spanned 10 consecutive days until a maximum of 50% was attained. On each day, the electrical resistance across all three samples was measured at a specific relative humidity level for that day. This process yielded a time- and humidity-dependent resistance profile R, used to evaluate sample stability against environmental moisture.


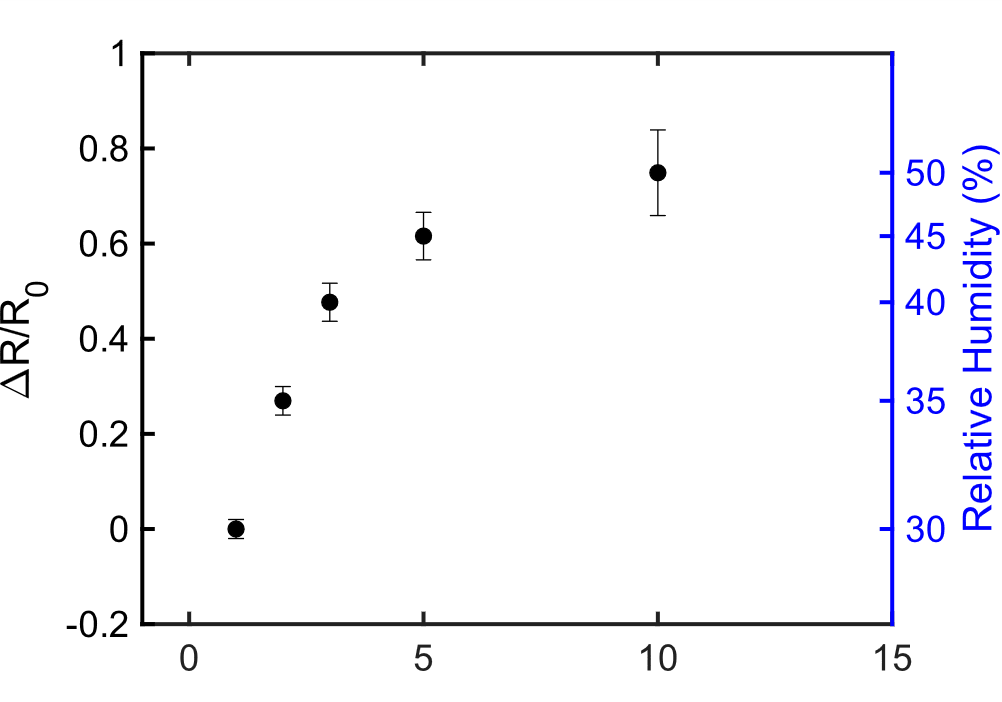


**Figure S19**: Stability and change in relative resistance (ΔR/*R*_0_) of the e-tattoo over ten days under increasing level of relative humidity (30% to 50%). As time and humidity increase, the relative resistance change (ΔR/R_0_) also rises, reflecting the device’s sensitivity to ambient moisture. Error bars show the SD across three separate samples, confirming measurement reliability.

**Figure S20**: Characterization of MAX phase, and MXene. (a) XRD analysis confirming the successful synthesis of Ti_3_C_2_T_x_ MXene from the Ti_3_AlC_2_ MAX phase, with the removal of the Al layer (2θ = 39°) and a shift in the (002) peak from 2θ = 9.5° to 7.44°. (b-c) SEM images showing the morphology of the MAX phase and exfoliated MXene after etching.


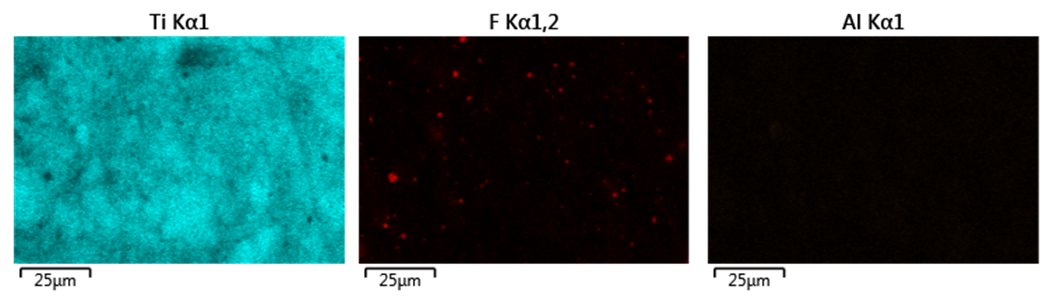


**Figure S21**: EDS elemental mapping of the MXene layer in the E-tattoo, (Left) Titanium (Ti) mapping, (Middle) Fluorine (F) mapping, indicating the presence of surface functional groups, (Right) Aluminum (Al) mapping, showing its localized presence, which may originate from the precursor materials or residual phases.

**Table S1**: Comparison of PVDF- MXene TENG with PVBVA based TENG

| S. No. | Materials | Methods | Output Voltage | Current (Current Density) | Power Density | Ref |
| --- | --- | --- | --- | --- | --- | --- |
| 1 | PVDF-TrFE/MXene (Ti_3_C_2_T_x_) | Electrospinning | 270 V | 140 mA/m^2^ | 4.02 W/m^2^ | [1] |
| 2 | PVDF/MXene (Ti_3_C_2_T_x_) composite (PMC) nanofibers | Electrospinning | 724 V | 163.6 µA | 11.213 W/m^2^ | [2] |
| 3 | PVA-PVDF  MXene (Ti_3_C_2_T_x_) | 3D printing and electrospinning | 1056V (95 % RH) | 36.6 μA | NA | [3] |
| 4 | PVA/MXene (Ti_3_C_2_T_x_) / Silk | Electrospinning | 117 V | NA | 1087.6 mW/m^2^ | [4] |
| 5 | PVDF/ V_2_CT*_x_* | Electrospinning | 124 V | 2.2 µA | 21.62 μW/m2 | [5] |
| 6 | PVDF (NF)/ (Ti_3_C_2_T_x_)/hBNNs/rGO | Electrospinning | 80 V | 250 nA/ cm^2^ | 22 mW/m^2^ | [6] |
| **7** | **PVBVA/MXene (Ti_3_C_2_T_x_)** | **Electrospinning** | **250 V** | **2.9 µA** | **250 mW/m^2^,** **25 µW/cm^2^** | **This work** |

Reference:

[1] S.M.S. Rana, M.T. Rahman, M. Salauddin, S. Sharma, P. Maharjan, T. Bhatta, H. Cho, C. Park, J.Y. Park, Electrospun PVDF-TrFE/MXene Nanofiber Mat-Based Triboelectric Nanogenerator for Smart Home Appliances, ACS Appl. Mater. Interfaces 13 (2021) 4955–4967. <https://doi.org/10.1021/acsami.0c17512>.

[2] T. Bhatta, P. Maharjan, H. Cho, C. Park, S.H. Yoon, S. Sharma, M. Salauddin, M.T. Rahman, S.S. Rana, J.Y. Park, High-Performance Triboelectric Nanogenerator Based on MXene Functionalized Polyvinylidene Fluoride Composite Nanofibers, Nano Energy 81 (2021) 105670. <https://doi.org/10.1016/j.nanoen.2020.105670>.

[3] X. Sun, L. Dong, Y. Liu, X. Li, J. Liu, N. Wang, Y. Liu, X. Li, D. Wang, S. Chen, Biomimetic PVA-PVDF-Based Triboelectric Nanogenerator with MXene Doping for Self-Powered Water Sterilization, Mater. Today Nano 24 (2023) 100410. <https://doi.org/10.1016/j.mtnano.2023.100410>.

[4] C. Jiang, C. Wu, X. Li, Y. Yao, L. Lan, F. Zhao, Z. Ye, Y. Ying, J. Ping, All-Electrospun Flexible Triboelectric Nanogenerator Based on Metallic MXene Nanosheets, Nano Energy 59 (2019) 268–276. <https://doi.org/10.1016/j.nanoen.2019.02.052>.

[5] M.S. Deepak, N.K. Das, S. Badhulika, V_2_CTx MXene Interspersed PVDF Electrospun Nanofibers Based Piezoelectric Nanogenerator for Self-Powered Electronic Devices and Mechano-Electrodeposition, J. Alloys Compd. 1007 (2024) 176426. <https://doi.org/10.1016/j.jallcom.2024.176426>.

[6] D. Sahoo, S. Sahoo, D. Nayak, S. Mohanty, R. Naik, Enhancing Triboelectric Performance Through 2D Nanofillers (MXene, hBN, rGO) in PVDF Nanofiber for Self-Powered Sensing, Adv. Mater. Technol. n/a (n.d.) e01141. https://doi.org/10.1002/admt.202501141.
